# Immune and stromal transcriptional patterns that influence the outcome of classic Hodgkin lymphoma

**DOI:** 10.1038/s41598-024-51376-1

**Published:** 2024-01-06

**Authors:** Victoria Menéndez, José L. Solórzano, Mónica García-Cosío, Ruth Alonso-Alonso, Marta Rodríguez, Laura Cereceda, Sara Fernández, Eva Díaz, Carlos Montalbán, Mónica Estévez, Miguel A. Piris, Juan F. García

**Affiliations:** 1 Translational Research, Fundación MD Anderson International España. Madrid, 28033 Madrid, Spain; 2 Pathology Department, MD Anderson Cancer Center Madrid, C/Arturo Soria, 270, 28033 Madrid, Spain; 3 Pathology Department, Hospital Universitario Ramón y Cajal, 28034 Madrid, Spain; 4 Pathology Department, IIS Hospital Universitario Fundación Jiménez Díaz, 28040 Madrid, Spain; 5 Hematology Department, MD Anderson Cancer Center Madrid, 28033 Madrid, Spain; 6 Center for Biomedical Network Research on Cancer (CIBERONC), ISCIII, 28029 Madrid, Spain

**Keywords:** Lymphoma, Cancer microenvironment

## Abstract

Classic Hodgkin lymphoma (cHL) is characterized by a rich immune microenvironment as the main tumor component. It involves a broad range of cell populations, which are largely unexplored, even though they are known to be essential for growth and survival of Hodgkin and Reed–Sternberg cells. We profiled the gene expression of 25 FFPE cHL samples using NanoString technology and resolved their microenvironment compositions using cell-deconvolution tools, thereby generating patient-specific signatures. The results confirm individual immune fingerprints and recognize multiple clusters enriched in refractory patients, highlighting the relevance of: (1) the composition of immune cells and their functional status, including myeloid cell populations (M1-like, M2-like, plasmacytoid dendritic cells, myeloid-derived suppressor cells, etc.), CD4-positive T cells (exhausted, regulatory, Th17, etc.), cytotoxic CD8 T and natural killer cells; (2) the balance between inflammatory signatures (such as IL6, TNF, IFN-γ/TGF-β) and MHC-I/MHC-II molecules; and (3) several cells, pathways and genes related to the stroma and extracellular matrix remodeling. A validation model combining relevant immune and stromal signatures identifies patients with unfavorable outcomes, producing the same results in an independent cHL series. Our results reveal the heterogeneity of immune responses among patients, confirm previous findings, and identify new functional phenotypes of prognostic and predictive utility.

## Introduction

The tumor microenvironment (TME) has been extensively studied and found to be a determinant in the tumorigenesis, evolution, and treatment sensitivity of many cancer types. One of the most representative models is classic Hodgkin lymphoma (cHL), whose main tumor tissue component comprises a rich and extensive immune microenvironment^1^. It is of note that Hodgkin and Reed–Sternberg (HRS) cells actively control and remodel the immune TME, and its constituents influence the treatment response^2^.

Previous studies have applied immunohistochemistry (IHC), microarrays, cytometry by time-of-flight, and NanoString assays for in situ cell phenotyping of the TME in cHL and have identified some important associations between the presence of certain immune cell types and clinical outcomes. For instance, it has been repeatedly described that a high frequency of macrophages is correlated with an unfavorable prognosis in cHL, especially M2-like macrophages^3^, based on the expression of single markers. Similarly, T cell subpopulations like cytotoxic T cells or regulatory T cells (Tregs) have also been associated with prognosis^4^. However, many more specific cell populations lack precise markers enabling identification, as is the case of M1-like macrophages^5^, Th17 cells^6^, myeloid–derived suppressor cells (MDSCs)^7^, etc.

NanoString is a novel high-throughput technology that requires minimal input (c. 100 ng RNA) from formalin-fixed, paraffin-embedded (FFPE) tissues, and produces results that are closely correlated with those of genomic expression arrays^8^. When combined with cell-deconvolution and gene-set enrichment analyses, new functionally relevant biomarkers and cellular components become accessible^9^. Each pathway and cell population is identified with a gene-set (GS), a collection of genes whose expression is enriched, offering the possibility of creating new GSs for different functional phenotypes and/or pathways.

We used NanoString gene-expression (GE) technology with a custom-adapted panel to identify the components that can explain the unresponsiveness of some patients to first-line treatment. Remarkably, several pathways, genes, and cell subsets related to stroma and specific immune cell populations, were enriched in patients who do not respond to therapy. The stromal microenvironment is already known to be crucial in lymphoma pathogenesis, but specific explanatory components were not identified yet. Here, we explore some of the mechanisms by which some elements of the cHL TME may influence prognosis, and their potential contribution to tumor immune-evasion strategies. As drawing conclusions from a cohort of 25 patients remains a delicate exercise, we used a confirmatory cohort from an RNA-seq study of 103 Hodgkin patients to verify our signatures.

## Methods

### Patient samples

Initially, diagnostic FFPE tumor biopsies and clinical data from 38 cHL patients were obtained from the participating institutions. IHC validations were performed on tissue microarrays (TMAs) containing an independent series of cHL FFPE biopsies.

### NanoString analysis and data preparation

GE profiling was performed with nCounter Technology (NanoString Technologies, Seattle, WA). Total RNA from diagnostic FFPE biopsies was isolated using the truXTRAC FFPE total NA kit (Covaris Inc., Woburn, MA), following the manufacturer instructions. Then, the NanoString nCounter® PanCancer Immune Profiling Panel with 30 extra custom genes (giving a maximum of 800 genes) was used. The complete gene list can be found in Supplemental Table 1. The resulting data were checked with nSolver software for quality control and normalization.

### Deconvolution analysis

Several GSs were selected from different sources and databases to encompass all relevant populations and features (Supplemental Table 2).

Enrichment scores for each GS and patient were computed independently by using GenePattern single-sample gene-set enrichment analysis (ssGSEA) tool. GE values for each sample were rank-normalized and an enrichment score was produced using the Empirical Cumulative Distribution Functions of the genes in the GS and remaining genes. Normalized enrichment scores were calculated by normalizing to the mean enrichment of random samples of the same size. The method employs random sampling of gene sets of the same size as the gene set being tested to assess significance and for normalization. Thus, a negative score means weaker relative activity in a sample compared with the background population, and a positive score means greater relative activity.

In addition, normalized bulk GE data were used to infer the estimated proportions of infiltrating immune cells using the CIBERSORTx tool (https://cibersortx.stanford.edu/). CIBERSORTx GS were drawn from GE values of the LM22 predefined signature matrix and individual cells from healthy humans (GSE118165, GSE135390, GSE128626, LM6, GSE129033, and GSE107011).

### Statistical and survival analysis

Each GE data from the panel, enrichment score from ssGSEA, and cell population frequency from CIBERSORTx was analyzed independently with pipelines developed in R (version 4.2.1-R Foundation for Statistical Computing, Vienna, Austria). A Shapiro–Wilk normality test was used before comparing means or medians. Then, significance (*p* < 0.05) was determined using Student unpaired samples t-tests when variables were normally distributed, and Kruskal–Wallis non-parametric tests otherwise. Univariate proportional hazards (Cox) regression analysis and division into tertiles and quartiles were employed for survival analysis, using Progression-Free Survival (PFS) as the endpoint. The Kaplan–Meier method was used to estimate patient survival and groups’ survival curves were compared using the log-rank test, using the cutoff defined by tertiles and quartiles.

### Validations

A bulk GE dataset (GSE132348) of 103 cHL patients from Gene-Expression Omnibus (GEO) was used to validate the results^10^. It was obtained using the same panel and methods as for the discovery dataset, without the 30 extra custom genes. After two cycles of ABVD, patients were classified following interim FDG-PET (iPET) response, where positive is equivalent to U (4 or 5 on the Deauville-five-point scale), and negative is equivalent to F. Early response to ABVD assessed with iPET is prognostic for cHL patients and has been used to identify GE profiles related to outcome^10^.

Selected markers were validated using IHC on TMAs constructed with duplicate 0.6-mm tissue cores from tumor-rich selected areas of archival FFPE tumor blocks. Primary antibodies were anti-PTPN2 (clone 2A1D1, Fisher Scientific), monoclonal anti-CD123 (clone 7G3, BD Pharmingen), anti-CD163 (clone 10D6, Leica Biosystems), anti-CD68 (clone 514H12, Leica Biosystems), anti-CD8 (clone 4B11, Leica Biosystems), and anti-granzyme B (GrB) (clone 11F1, Leica Biosystems). Staining was done with the BOND RX automated Stainer system (Leica Biosystems). IHC counts were manually scored by one of the authors (J.L.S.R.). Finally, the most significant variables from ssGSEA that were correlated with a bad prognosis were included in a prognostic validation model.

### Helsinki statement

All procedures were performed in accordance with relevant guidelines. Samples and data were collected through the MD Anderson Cancer Center Madrid Biobank, following the technical and ethical procedures of the Spanish National Biobank Network, including anonymization processes.

### Informed consent

Informed consent was obtained from all subjects and/or their legal guardian(s).

### Ethics committee

Approval for this study was obtained from the institutional review board (CEIm H. Ramón y Cajal, ref. 445/22).

### Conference presentation

Presented in abstract form at the 3rd PhD Symposium in Health Sciences and Biomedicine, UAM, Madrid, Spain. June 3rd, 2022

## Results

### Patient characteristics and immune fingerprints

After filtering, our dataset contained 25 patients (86.1% overall performance). Patient outcome was considered favorable (F) when their PFS was higher than 2 years; otherwise, the outcome was considered unfavorable (U). All cases were treated with standard adriamycin-based protocols, mostly ABVD. The cHL cohort was enriched in U cases (56%) to enable the study of the factors that confer a poor prognosis. Their clinicopathological characteristics are summarized in Supplemental Table 3 and detailed in Supplemental Table 9. There were no significant differences in any clinical variable between groups U and F, except gender and response to the first line of therapy, as has been repeatedly reported.

Conventional tools for raw GE data exploration and visualization identify individual genes with differential expression between clinical phenotypes (U vs F and relapsed vs non-relapsed patients, Supplemental Fig. 1). Consistent with previous findings, we identified *VEGFA*, *SPP1*, *TREM1*^10^,* NOS2*^11^,* S100A8*, *S100A9*^12^,* LILRB2*^13^, and *LYZ*^14^ genes (among others) as being enriched in relapsed or U patients. However, the information derived from analyses using individual genes is limited and diffuse and does not allow conclusions to be drawn about biological significance, so we added extra deconvolution steps to facilitate phenotypic characterization with the ssGSEA and CIBERSORTx tools.

**Figure 1:**
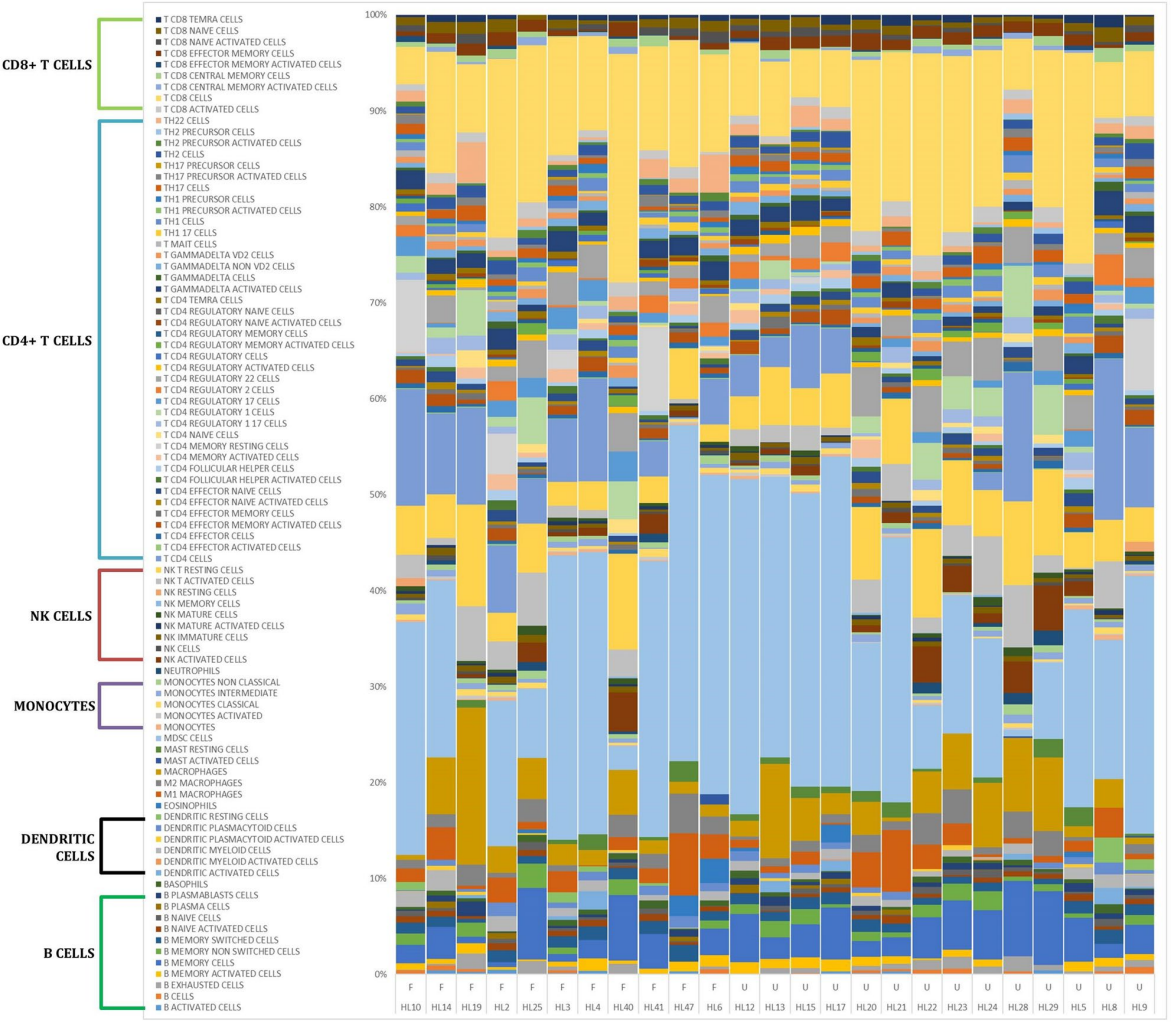
Cell specificity achieved in CIBERSORTx. Representation of the fingerprint of cell abundance measured by CIBERSORTx in each patient. F and U patients are shown on the left and right, respectively.

ssGSEA was used to estimate the enrichment scores for cell populations and pathways. A GS enrichment score represents the activity level of the biological process in which its members are coordinately upregulated or downregulated. Unlike with the traditional GSEA tool, we could accurately determine the enrichment score for each component for every patient, allowing more powerful statistical and survival analyses. Likewise, using CIBERSORTx, we were able to estimate the abundance of 89 distinct functional subpopulations, including unexplored specialized cell types with extraordinary sensitivity. Initial analyses using that tool for mapping all the cHL profiles revealed remarkable heterogeneity among patients (Fig. 1), highlighting the importance of individual immune fingerprints of the TME that seem to trigger distinct immune cell responses^15^.

### F and U patients differ in inflammatory balance and MHC molecule distribution

**Figure 2:**
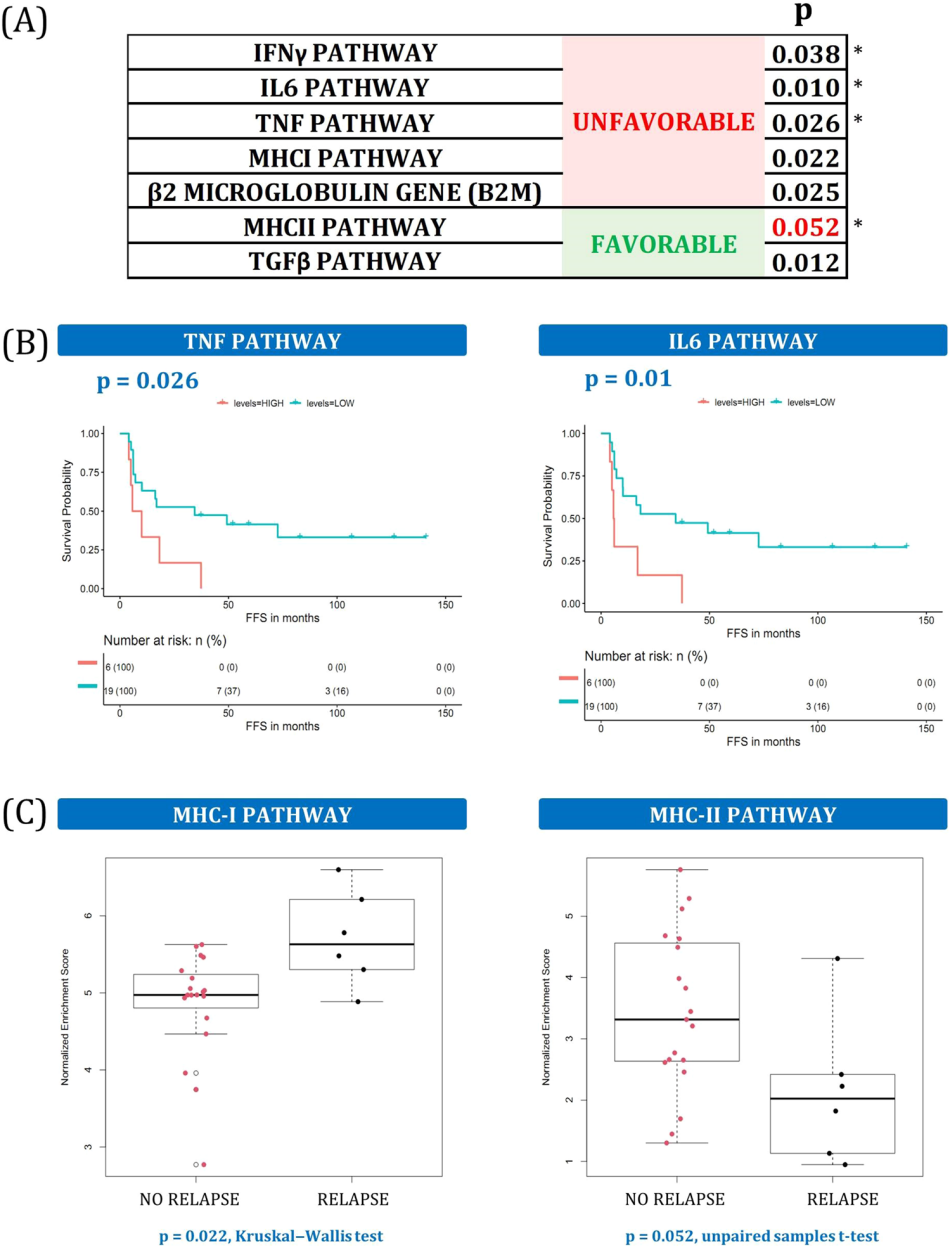
Inflammation balance and MHC molecules. (**A**) Summary of inflammation and MHC variables associated with outcome. * indicates that the same variable was also statistically significant in the validation dataset. More details can be found in Supplemental Table 4 and Supplemental Fig. 2. (**B**) Kaplan–Meier plots of TNF pathway and IL6 enrichment scores, dichotomized into high and low groups. (**C**) Boxplots of MHC-I and MHC-II pathway-enrichment scores for non-relapsed and relapsed patient groups.

TGF-β pathway has yielded controversial results in non-Hodgkin Lymphoma, showing pro- or anti-tumorigenic behavior, depending on the circumstances^16^. In our cHL cohort, the TGF-β pathway was enriched in responders, whereas the IFN-γ, TNF, and IL-6 signaling pathways predominated in poor responders (Fig. 2A,B). As greater inflammation significantly contributes to the creation of a U TME in cHL^17^ (represented by IFN-γ, TNF and IL-6), the anti-inflammatory effects of the TGF-β pathway may counteract their pro-inflammatory effects.

An increase in the expression of MHC-II molecules confers a better prognosis in cHL and in other cancers, while MHC-I expression is not predictive of complete remission^18^. Consistently, MHC-II pathway upregulation was beneficial to cHL survival in our cohort, while the MHC-I pathway was associated with the opposite effect (Fig. 2C). The *β2-Microglobulin* gene belongs to the MHC-I pathway cascade and behaved identically (Supplemental Table 4).

### Myeloid cells strongly influence prognosis

**Figure 3:**
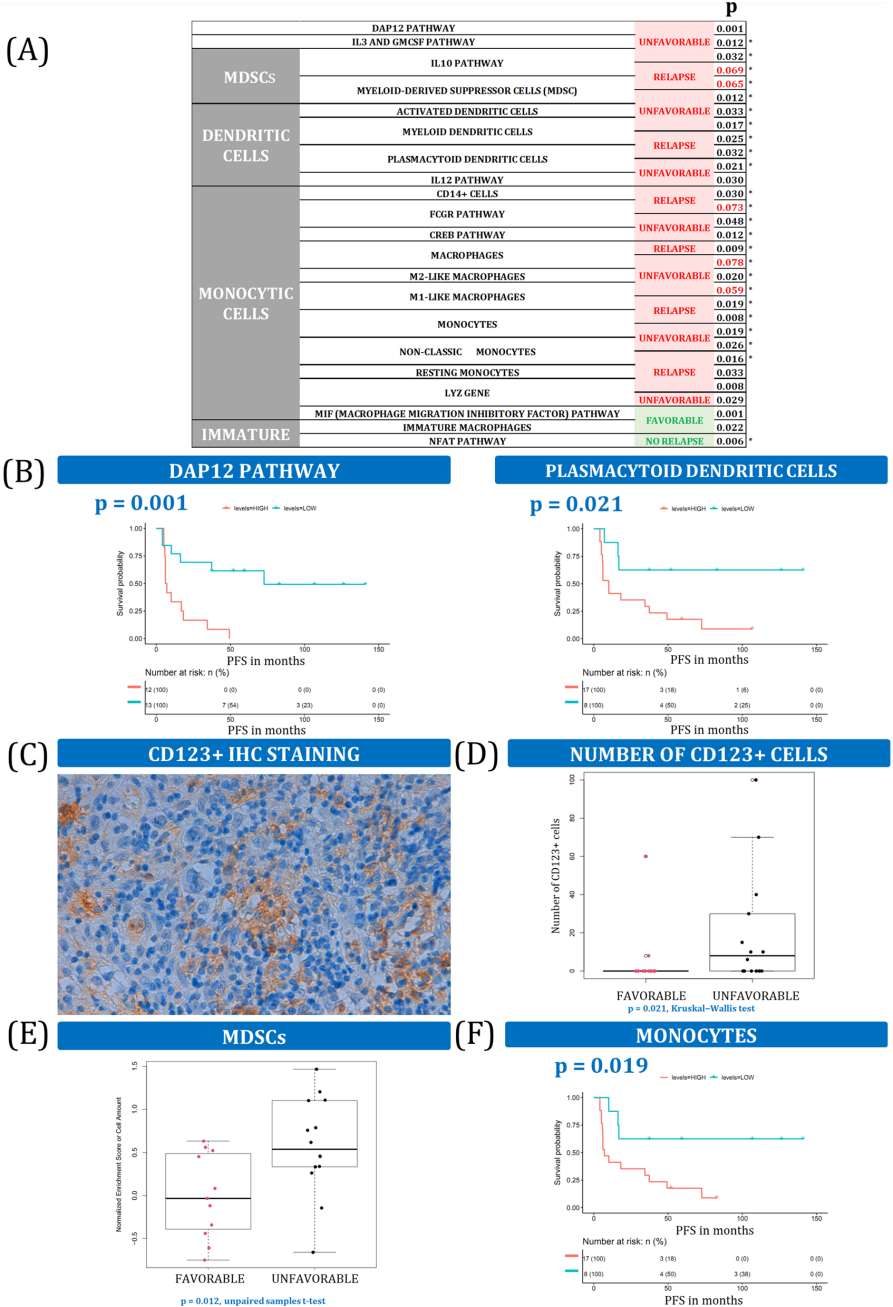
Myeloid cells. (**A**) Summary of myeloid cells and related variables associated with outcome. * Indicates that the same variable was also statistically significant in the validation dataset. More details can be found in Supplemental Table 5 and Supplemental Fig. 3. (**B**) Kaplan–Meier plots of DAP12 pathway and plasmacytoid dendritic cell enrichment scores dichotomized into high and low groups. (**C**) IHC image of anti-CD123 antibody staining (original magnification 200x) in the validation cohort. (**D**) Boxplot of the number of CD123 + cells in F and U patient groups in the validation cohort. (**E**) Boxplot of MDSC cell enrichment scores for F and U patients. (**F**) Kaplan–Meier survival curve for monocyte cell frequencies, by high- and low-level groups.

Some GSs related with myeloid cells, such as the IL-3 and GMCSF pathways, were upregulated in cHL patients with bad prognosis (Fig. 3A), as seen before^19^. DAP12 pathway expression was also enriched in U cHL patients, which promotes myeloid cell differentiation and is associated with mature stages of myeloid development (Fig. 3B)^20^. Conversely, markers that suffer downregulation upon myeloid differentiation, such as the NFAT pathway^21^, were enriched in patients who respond to the therapy. This is aligned with the F prognosis associated with the immature macrophages in our discovery dataset.

Supporting the idea that myeloid cells contribute to an U TME in cHL, our results show that dendritic cells (DCs) are also enriched in patients who relapse or do not respond to the first-line therapy. These cell populations include myeloid, plasmacytoid, activated DCs, and their related pathway IL-12 (Fig. 3B), consistent with other similar findings^14^. Furthermore, we used an independent cohort of cHL patients to validate the results by IHC using TMAs (N = 28 valid cases). The IL-3 receptor alpha chain (IL3Rα, CD123) is a classic marker of plasmacytoid DCs. We found that a higher number of CD123-positive cells was associated with lower PFS (Fig. 3C,D), confirming the results of the GE analysis.

Interestingly, MDSCs^22^ and related IL-10 pathway^23^ were associated with adverse outcomes (Fig. 3E). The same pattern was observed by other authors^3^ in monocytic cells, including CD14-positive cells, monocytes, and macrophages (Fig. 3F). We also confirmed that CD68 and CD163 markers are strongly associated with worse outcomes by IHC analyses (N = 43, *p* < 0.001). Within those monocytic cell populations, we were able to depict accurately the cell phenotypes that were most highly enriched in bad responders (monocytes, M1-like, M2-like, and activated macrophages), the most relevant pathways (FCGR and CREB), and some relevant genes (Supplemental Table 5).

### Cytotoxic cell maturation status affects cHL TME behavior

**Figure 4:**
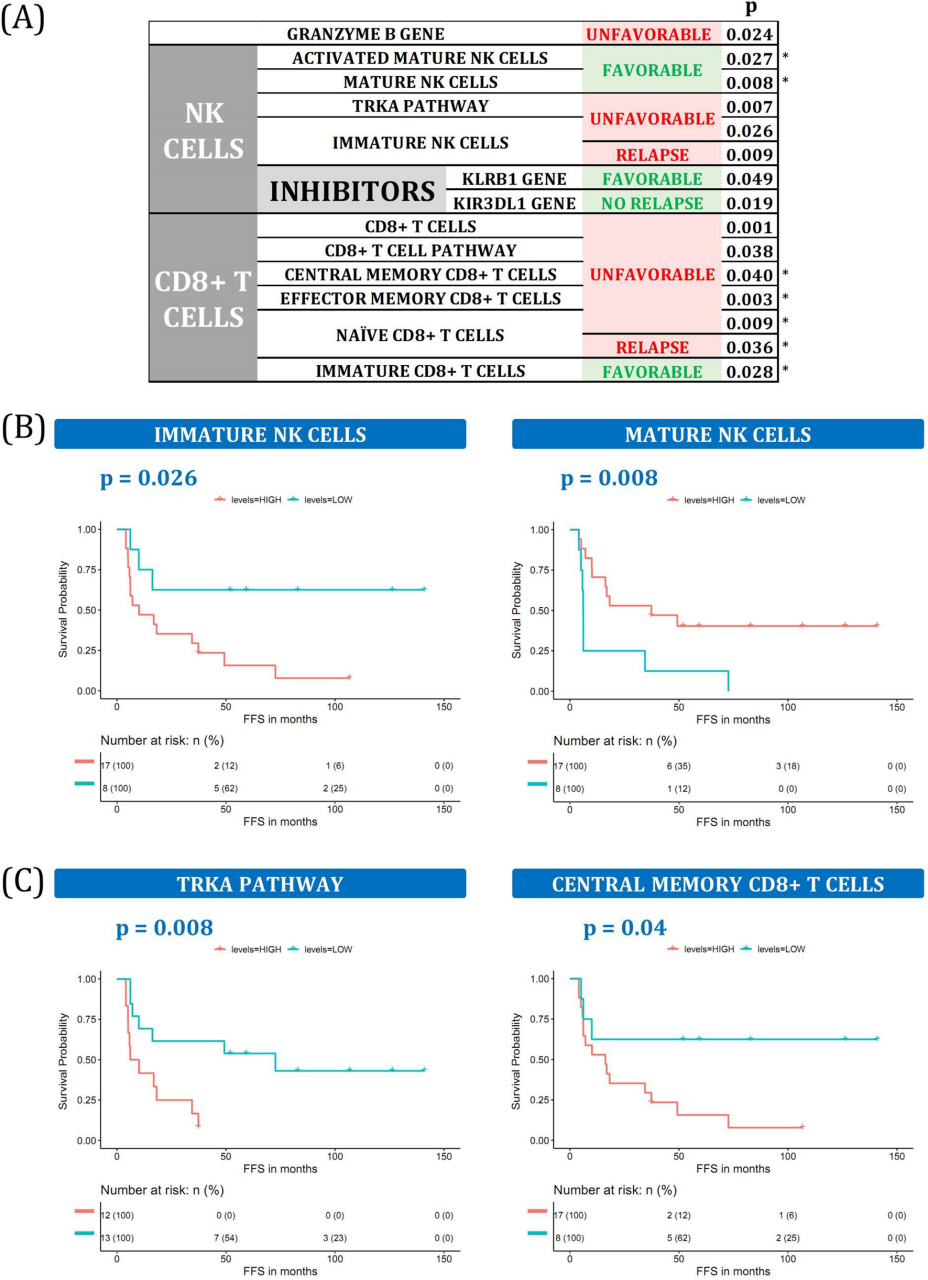
Cytotoxic cells. (**A**) Summary of cytotoxic cells and related variables associated with outcome. * indicates that the same variable was also statistically significant in the validation dataset. More details can be found in Supplemental Table 6 and Supplemental Fig. 4. (**B**) Kaplan–Meier plots of TRKA pathway enrichment scores and central memory CD8 + T cell amounts dichotomized in high and low groups. (**C**) Kaplan–Meier plots of immature and mature NK cells dichotomized into high and low groups.

Some genes associated with cytotoxic T cells or natural killer (NK) cells are known to be associated with a worse prognosis in cHL^24^, along with CD8 T cells that express markers such as TIA-1 or GrB^25^. Accordingly, IHC analyses in our independent cohort revealed that worse outcomes were associated with CD8 and GrB markers (N = 31, *p* < 0.01). However, results about the general contribution of cytotoxic cells to survival are contradictory^26^. We confirmed the hypothesis that their influence on survival depends on their differentiation or maturation status by focusing on specific cytotoxic phenotypes (Fig. 4A).

Some regulators of NK cell function, such as the *KLRB1* and *KIR3DL1* genes, are associated with the absence of relapse and an increased PFS in cHL, consistent with previous findings in many cancer types^27^. While mature and activated mature forms of NK cells seem to have the same effect, some general NK cell markers (*GNLY, CD244*, and *CD48* genes, TRKA pathway) and their most immature forms are detrimental to survival (Fig. 4B,C). Likewise, CD8 T cells can affect the prognosis depending on their maturation level, but with converse implications. Stem cell memory CD8 T cells, which are the most immature phenotype, are enriched in F patients, whereas more mature forms are associated with lower PFS (naïve, TEMRA, effector memory, and central memory CD8 T cells), and with CD8 T cell signaling (Fig. 4C and Supplemental Table 6).

### Stroma is associated with poorer survival and more frequent relapse

**Figure 5:**
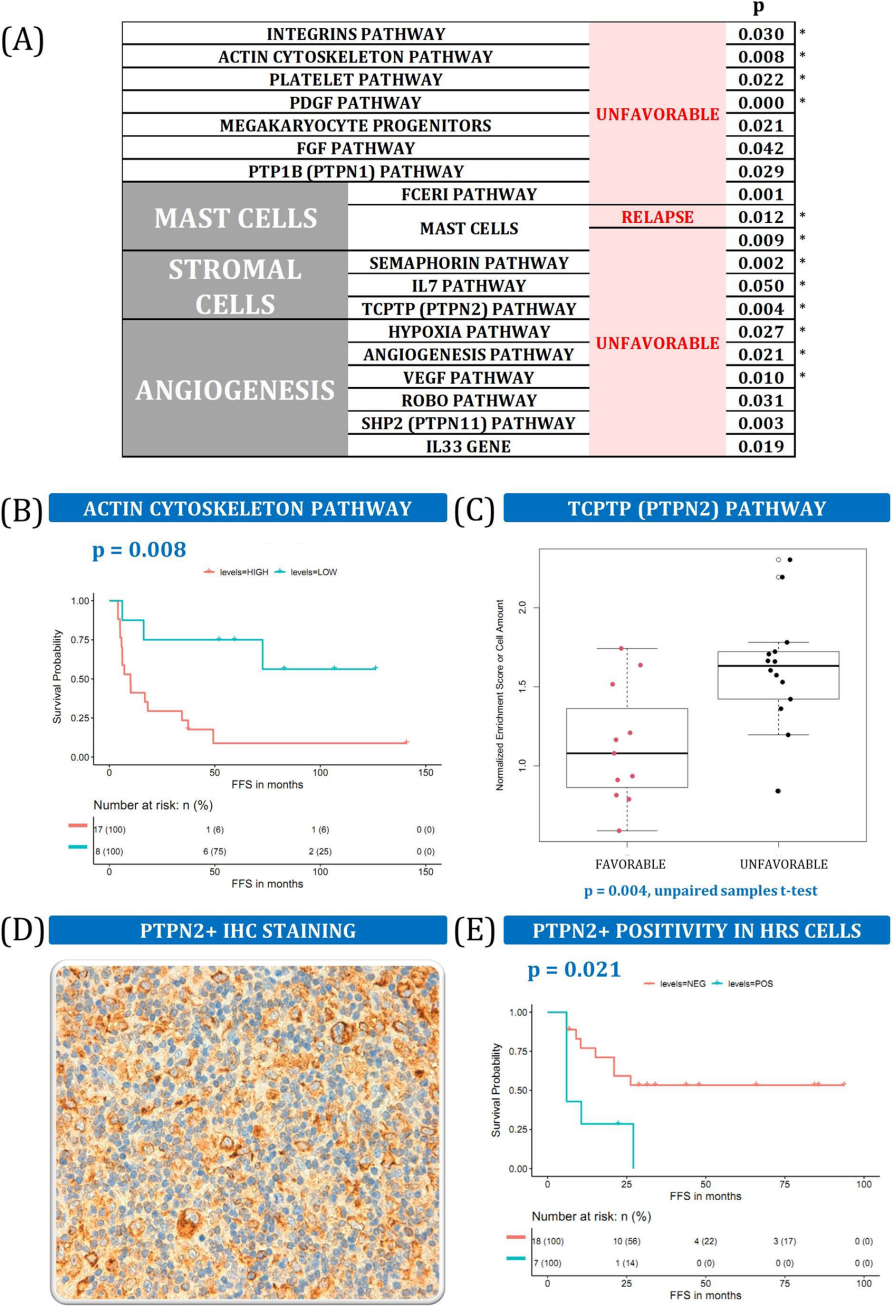
Stromal components. (**A**) Summary stromal components associated with a bad outcome. * indicates that the same variable was also statistically significant in the validation dataset. More details can be found in Supplemental Table 7 and Supplemental Fig. 5. (**B**) Kaplan–Meier plot of actin enrichment scores dichotomized into high and low groups. (**C**) Boxplot of TCPTP pathway enrichment scores for F and U patients. (**D**) IHC image of anti-PTPN2 antibody staining (original magnification 200x). (**E**) Kaplan–Meier survival curve split into positive and negative anti-PTPN2 staining for HRS cells.

Several stromal components were associated with a bad outcome in our cohort (Fig. 5A). Even though the adverse influence of stroma in cHL had already been documented^28^, it lacked a more detailed and precise description. Stromal factors that were enriched in U patients comprised a broad range of genes, pathways, and cell types involved in the actin cytoskeleton, integrins from the extracellular matrix (ECM), platelet activity, or fibroblast growth factor (FGF) (Fig. 5B). Several cell populations also play an important role, like mast cells, as documented elsewhere^29^. They are directly related to angiogenesis, whose components showed strong associations with the response to the first-line treatment (Supplemental Table 7). Those include pathways such as hypoxia, ROBO, SHP2, and vascular endothelial growth factor (VEGF) signaling. Finally, stromal cells demonstrated a significant influence on the outcome of the disease through semaphorin and TCPTP pathways, as well as IL-7 signaling, which is detrimental to cHL patients and copiously produced by stromal cells (Fig. 5C).

This stromal signature is not correlated with the histological subtype of the cases (nodular sclerosis cHL *vs.* others), and the histological subtype is not significantly related to clinical outcome (data not shown), which indicates that this stromal signature contains functional information beyond the mere morphological appearance of the tissues.

TCPTP phosphatase, also known as PTPN2, was used to validate the stromal findings by IHC analysis in an independent cohort. In mice, TCPTP is a negative regulator of colony-stimulating factor 1 signaling and macrophage differentiation^30^. Total stained cells were counted, distinguishing expression by HRS cells or TME cells. As expected, PTPN2-positive HRS cells were significantly enriched in U patients (Fig. 5D,E).

### The CD4 T cell spectrum has contrasting prognostic implications

**Figure 6:**
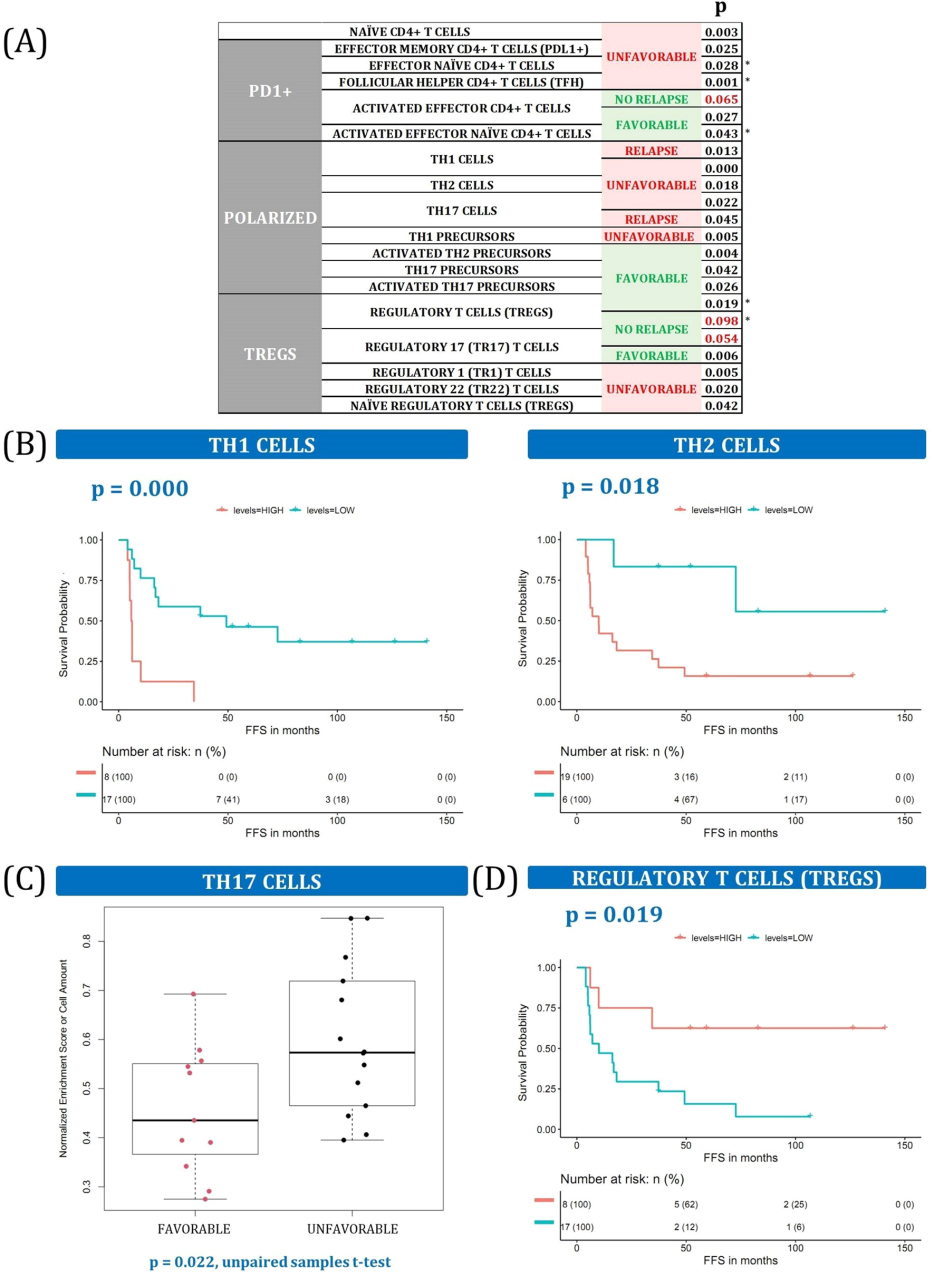
CD4 + T cells. (**A**) Summary of CD4 + T cells associated with outcome. * indicates that the same variable was also statistically significant in the validation dataset. More details can be found in Supplemental Table 8 and Supplemental Fig. 4. (**B**) Kaplan–Meier plots of Th1 and Th2 cell frequencies dichotomized into high and low groups. (**C**) Boxplot of Th17 cell frequencies in F and U patients. (**D**) Kaplan–Meier plots of Tregs dichotomized into high and low groups.

We found a dual effect in survival involving a large repertoire of CD4 T cell phenotypes (Fig. 6A). Inside the PD1-positive CD4 T groups, effector memory, effector naïve, and follicular helper CD4 T cells (Tfh) were found to be detrimental to cHL survival, whereas activated effector CD4 T cells, both naïve and total, were F. Polarized CD4 T cells encompass another important compartment that influences the outcome in cHL, and several of its components had already been studied. Consistent with previous findings, the presence of Th1^31^, Th2, and Th17^32^ cells was correlated with worse PFS (Fig. 6B,C). Strikingly, we found that their precursors also play an important role in the disease, whereby Th1 precursors are the only bad prognostic subset. Instead, levels of total and activated Th2 and Th17 precursors were associated with higher PFS and fewer relapses (Supplemental Table 8).

Finally, we studied the regulatory T cell compartment, confirming previous findings like the association between FOXP3-positive Tregs and an F prognosis^4^ (Fig. 6D). Surprisingly, Tr17 (T regulatory 17) cells had the same effect, whereas other Tregs conferred worse outcomes. Among them, the association between Tr1 cells and worse survival had already been documented^23^, and we noticed that the Tr22 and naïve Treg cell populations had the same bad influence.

### Validation model

**Figure 7:**
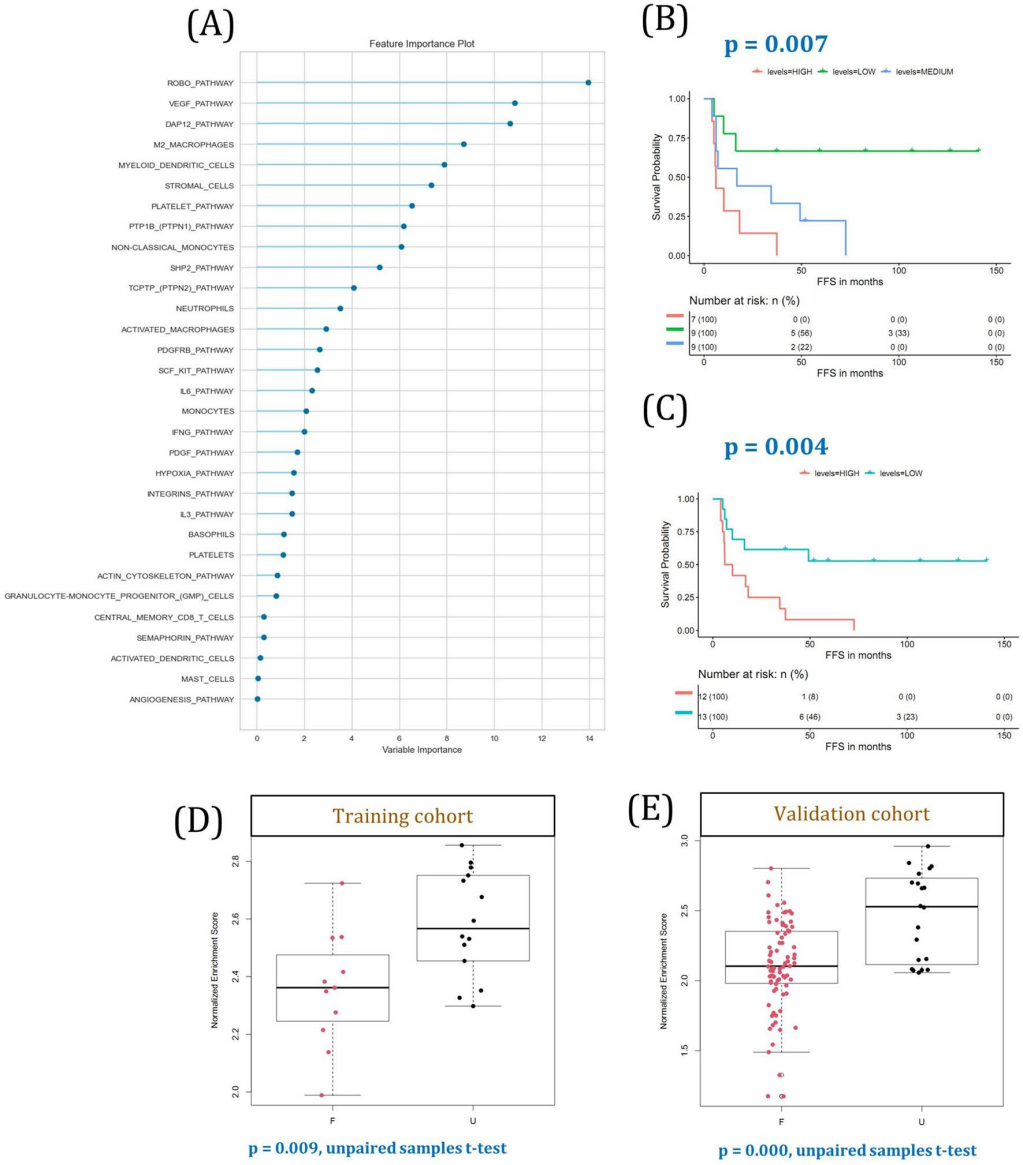
Validation model with cell and pathway enrichment scores. (**A**) 31 variables were included in the model, of which 13 are cell types and 18 are signaling pathways. Those variables are represented in decreasing order, as calculated with the pycaret Python package. (**B**) The score was split into tertiles and plotted using the Kaplan–Meier method. (**C**) Patients were dichotomized at 50% into high and low scores and plotted as Kaplan–Meier survival curves. (**D**) Boxplots of training and validation cohorts are shown comparing F versus U cHL patients. Training cohort, *p* = 0.009; validation cohort, *p* = 0.0001.

We created a predictive model to categorize cHL patients using the most critical prognostic ssGSEA variables, including cell populations and signaling pathways. The Shapiro–Wilk test results revealed that the vast majority were non-normally distributed variables, so, for each patient, we calculated the median of the enrichment scores for each component and computed the mean to obtain the model score. Some of the most important constituents of the model were the ROBO, VEGF, and DAP12 pathways, and M2-like macrophages, myeloid DCs, and stromal cells (Fig. 7A). Statistical analyses applied to the model score revealed a correlation between its value and survival. The group of patients with the highest score had a significantly worse prognosis, as shown by the tertiles analysis (Fig. 7B), by 50% dichotomization (Fig. 7C), and by boxplot analysis, both in the training dataset (Fig. 7D) and the validation cohort (Fig. 7E). Finally, the ROC curve method was applied to obtain the cutpoint between high-risk and low-risk patients, resulting in the correct classification of 76% of the cases in the training cohort and 81% when the model was applied to the validation cohort (Supplemental Fig. 7A,B).

## Discussion

cHL is unique among hematopoietic cancers in that its tumor mass is primarily composed of immune cells, which are mostly recruited by the much rarer neoplastic HRS cells. These cells not only engage and recruit nonmalignant immune cells to shape a distinctive TME, but also establish complex crosstalk with the components of the immunological ecosystem in favor of immunosuppressive elements more favorable to tumor growth.

Previous work seeking to describe TME components mainly relied on specific cell population identification using single IHC markers. Evidence indicates that most labels based on limited phenotypic markers used to describe immune populations are oversimplistic. For instance, the identification of macrophages or cytotoxic cells as unitary entities based on the expression of CD68^3^ or GrB^33^ can never allow the recognition of the complexity of cell differentiation and activation states that are inherent to the immune system, which could have contrary effects on immunotolerance.

Microarray signatures have also provided relevant pathophysiology data and prognostic information^34^. However, the clinical integration of these complex technologies has been proven to be limited. Also, current and emerging approaches to study the cHL TME rely on the simultaneous detection of markers using multiplexed fluorescence microscopy methods^35,36^, which allow a more detailed description of cell phenotypes and spatial distribution, but still based on a limited set of predefined markers.

Here, we show that using NanoString technology in FFPE tissues is feasible and, when coupled with deconvolution bioinformatics, can provide a similar level of performance as flow cytometry or physical cell sorting. This results in a significant gain of signal and resolution, in particular from scarce cell types, and allows more sensitive survival analyses and tumor subtype predictions^37^.

Our results confirm previous findings concerning the major clinical relevance of monocytic groups and T cells. Several monocyte-macrophage populations are correlated with worse clinical outcomes, with contrasting effects on survival depending on the balance between the number of cytotoxic T cells (CD8 and NK cells) and CD4 T lymphocyte phenotypes. Functional states of cells can be defined much more accurately following our approach, which enables the study of such specific subgroups as immature and mature T cells, or M1-like or M2-like macrophages, the identification of cells without specific IHC markers (such as Th17, Tr1, Tr2, MDSCs, etc.) and quantification of rare cell populations (granulocytic or monocytic MDSCs, Tfh cells, or others).

Our findings are consistent with a predominance of T cell exhaustion in the cHL TME, which was previously proposed as an additional base of immunotolerance. In fact, Cader et al.^38^ compared tissue samples from seven primary cHL patients with ten reactive lymph nodes or tonsils by mass cytometry and found that, in contrast to normal lymphoid tissues, cHL showed expanded populations of Th1-polarized Tregs and more differentiated CD4 Th1-polarized effector cell populations. Th1 Tregs expressed little or no PD1, whereas the effector Th1 cells were PD1-positive. Differential PD1 expression and functional Th1-polarized CD4 Tregs may represent exhausted T effectors as complementary mechanisms of immunosuppression^39^.

Here we identify several inflammatory response-related pathways, including MHC-I and MHC-II, IFN-γ, IL-6, TNF and TGF-β signaling, that are fundamental to the significance of cytokine-mediated immune-stroma crosstalk. Thus, IL-6 RNA transcript, IL-6 protein, and IL-6 receptors have been detected in HRS cells, leukocytes, and stromal cells in the cHL TME^40^. In fact, HRS cells produce IL-6 to polarize T cells to the Th17 phenotype, and Th17 cells recruit myeloid cells and promote angiogenesis. Accordingly, IL-6-positive leukocytes in the cHL TME are correlated with inferior survival^41^. Another example is TNFα, whose neutralization in the supernatants derived from cHL cell lines significantly inhibited myeloid DC maturation^42^. IFN-γ is an additional factor that prevents primary tumor development and shapes immunogenicity^43^. Moreover, interferon signaling in tumor cells increases the resistance to immune checkpoint blockade^44^.

Tr1 cells secrete IL-10, which is also associated with bad outcomes, and locates where there are MHC-II deficiency and higher levels of IL-6, the latter being known to induce Tr1 cells. Furthermore, TEMRA T cells can be highly enriched among CD4-positive cytotoxic T lymphocytes, which can exhibit GrB-mediated tumor cytotoxicity in an MHC-II-restricted manner^39^.

The most striking observation is the prognostic relevance of signatures and cell populations related to stromal functions and dynamics, even though they had already been reported in classic and less detailed reports using microarrays in DLBCL and cHL^14,45^. These stromal signatures are characterized by the upregulation of pathways involved in fibroblast activation, angiogenesis, and ECM remodeling^46^.

Cancer-associated fibroblasts (CAFs) constitute a significant cellular fraction of the TME that engages in dynamic crosstalk with cancer cells, infiltrating TAMs and other stromal elements^47^. They form a heterogeneous collection of activated fibroblasts secreting a wide repertoire of molecules that regulate tumor development and progression, inflammation, drug resistance, metastasis and recurrence. Cytokines produced by the malignant HRS clone probably recruit resident fibroblasts to a CAF precursor state and, ultimately, to a “mature” CAF phenotype^48^. Such dialogue between CAFs and cancer cells probably promotes cellular plasticity, tumor progression, maintenance of cancer cell stemness, metabolic reprogramming, ECM remodeling, and metastasis^49^.

It has been shown that extracellular vesicles (EVs) collected from HRS cells were internalized by fibroblasts and triggered their migration capacity. EV-treated fibroblasts are characterized by an inflammatory phenotype, upregulation of alpha-smooth muscle actin (α-SMA, a well-known marker of CAFs), and the expression of proangiogenic factors^50^. Consistent with our results, a newly identified subset of PDGFRα FAP double-positive CAFs has prognostic consequences for survival in cHL patients, and shows enrichment of focal adhesion and ECM genes^51^.

Our stromal signature also includes transcriptional data linked with angiogenesis, VEGF, and platelet functions. These results strengthen evidence of dynamic crosstalk between tumor-associated endothelial cells and cancer cells regulating tumor aggressiveness^52^. We have also reported that COX-2 expression was an unfavorable prognostic factor in cHL^46^. Dual inhibition of COX-2 and VEGF pathways reduced tumor metastasis and extended overall survival in several in vivo cancer models^53^.

Also, tumor cells and platelets maintain a complex, bidirectional interaction in the blood and the TME in several models^54^. The concept of “tumor-educated platelets” (TEPs) refers to the fact that tumor cells induce platelet EVs generation, granule release, and phenotypic changes by increasing the secretion of pro-angiogenic proteins, such as VEGF^55^, and by modifying stromal elements. In addition, platelets help cancer sustain proliferative signaling, resist cell death, and induce tumor angiogenesis^56^. Some rationale for antiplatelet agents as therapy for different cancers has recently been proposed^57^.

Functional phenotypes can be integrated in an outcome-predictive model reproducible in an independent series. We^34^ and others^10,24^ had previously proposed genetic predictive models for cHL, but these were based only on the selection of individual genes. Our approach allows integration of information from cell abundancies, immune signatures, and relevant pathways. Furthermore, most classic models rely on microarray technologies^24^ or digital GE profiling^58^, which are largely based on cell scores derived from GE data and have only been validated for some clinical subsets^58,59^. Our series are too short to allow us to propose a definitive model, since the number of variable combinations is very high. Similar pipelines in larger and more homogeneous series would be worthwhile, probably based on prospective and longitudinal samples from clinical trials.

Since 25–30% of cHL patients with advanced stages do not respond to standard therapies, improved risk stratification is needed to refine first-line treatments and to rationalize the use of immunotherapeutic agents. Studying the immunoregulatory role of hematopoietic and non-hematopoietic stromal cells in cHL and their intricate interactions with the lymphoma cells should help in designing next-generation immunotherapies and combination treatment strategies to overcome the complex TME-driven immune suppression. In the present study, we used a simple technology that allows in-depth dissection of cHL TME, annotate cellular and molecular heterogeneity, and identify functional phenotypes of prognostic and predictive utility.

## Supplementary Information

Supplementary Information.

## Data Availability

GE data are available at GEO under accession number GSE227589.
