# Author Correction: Immune and stromal transcriptional patterns that influence the outcome of classic Hodgkin lymphoma

**DOI:** 10.1038/s41598-024-78993-0

**Published:** 2024-11-11

**Authors:** Victoria Menéndez, José L. Solórzano, Mónica García-Cosío, Ruth Alonso-Alonso, Marta Rodríguez, Laura Cereceda, Sara Fernández, Eva Díaz, Carlos Montalbán, Mónica Estévez, Miguel A. Piris, Juan F. García

**Affiliations:** 1 Translational Research, Fundación MD Anderson International España. Madrid, 28033 Madrid, Spain; 2 Pathology Department, MD Anderson Cancer Center Madrid, C/Arturo Soria, 270, 28033 Madrid, Spain; 3 Pathology Department, Hospital Universitario Ramón y Cajal, 28034 Madrid, Spain; 4 Pathology Department, IIS Hospital Universitario Fundación Jiménez Díaz, 28040 Madrid, Spain; 5 Hematology Department, MD Anderson Cancer Center Madrid, 28033 Madrid, Spain; 6 Center for Biomedical Network Research on Cancer (CIBERONC), ISCIII, 28029 Madrid, Spain

Correction to: *Scientific Reports* 10.1038/s41598-024-51376-1, published online 06 January 2024

The original version of this Article contained an error in the Acknowledgements section.

“This work was supported by Instituto de Salud Carlos III (ISCIII), co-funded by European Regional Development Fund/European Social Fund (PI19/00083 and PI22/00556), Ministerio de Economía, Industria y Competitividad (MINECO), Spain (CIBERONC CB16/12/00291), Dirección General de Universidades e Investigación Consejería de Educación e Investigación de la Comunidad de Madrid (B2017/BMD-3778), Spain, and a Roche Foundation Research Grant. V.M. is a recipient of a PFIS predoctoral contract from ISCIII-AES-2020 (FI20/00184).”

now reads:

“This work was supported by Instituto de Salud Carlos III (ISCIII), projects PI19/00083 and PI22/00556 (co-funded by the European Union), Ministerio de Economía, Industria y Competitividad (MINECO), Spain (CIBERONC CB16/12/00291), Dirección General de Universidades e Investigación Consejería de Educación e Investigación de la Comunidad de Madrid (B2017/BMD-3778), Spain, and a Roche Foundation Research Grant. V.M. is a recipient of a PFIS predoctoral contract from ISCIII-AES-2020 (FI20/00184).”

The original Article has been corrected.

