# Supplementary material for: Immune and stromal transcriptional patterns that influence the outcome of classic Hodgkin lymphoma
Source: Sci Rep. 2024 Jan 6;14:710. doi: 10.1038/s41598-024-51376-1 (PMC10771441; doi:10.1038/s41598-024-51376-1)
Supplement: Supplementary file 1 — Supplementary Information. [file 41598_2024_51376_MOESM1_ESM.docx]

**Immune and stromal transcriptional patterns that influence the outcome of classic Hodgkin Lymphoma**

**Victoria Menéndez, José L. Solórzano, Mónica García-Cosío, Ruth Alonso-Alonso, Marta Rodríguez, Laura Cereceda, Sara Fernández, Eva Díaz, Carlos Montalbán, Mónica Estévez, Miguel A. Piris, and Juan F. García.**

**Supplemental Table 1.** **Genes used in the analysis.** The custom genes specific to Hodgkin’s Lymphoma are included with the NanoString nCounter® PanCancer Immune Profiling Panel genes, representing up to 800 genes in total.

| A2M | ACVR1C | ADAM12 | ADGRE1 | ADM | ADORA2A | AKT1 | ALDOA | ALDOC | ALK | ANGPT1 |
| --- | --- | --- | --- | --- | --- | --- | --- | --- | --- | --- |
| ANGPT2 | ANGPTL4 | ANLN | APC | APH1B | API5 | APLNR | APOE | APOL6 | AQP9 | AREG |
| ARG1 | ARG2 | ARID1A | ARNT2 | ATF3 | ATM | AXIN1 | AXL | B2M | BAD | BAMBI |
| BATF3 | BAX | BBC3 | BBS1 | BCAT1 | BCL2 | BCL2L1 | BCL6B | BID | BIRC3 | BIRC5 |
| BLK | BLM | BMLF1 | BMP2 | BNIP3 | BNIP3L | BRCA1 | BRCA2 | BRD3 | BRD4 | BRIP1 |
| BTLA | BZLF1 | C1QA | C1QB | C2 | C5 | C5AR1 | C7 | CASP1 | CASP3 | CASP8 |
| CASP9 | CBLC | CCL13 | CCL14 | CCL18 | CCL19 | CCL2 | CCL20 | CCL21 | CCL22 | CCL3/L1 |
| CCL4 | CCL5 | CCL7 | CCL8 | CCNA1 | CCNB1 | CCND1 | CCND2 | CCND3 | CCNE1 | CCNO |
| CCR2 | CCR4 | CCR5 | CD14 | CD163 | CD19 | CD1C | CD1a | CD2 | CD207 | CD209 |
| CD23 | CD244 | CD247 | CD27 | CD274 | CD276 | CD28 | CD300A | CD33 | CD36 | CD38 |
| CD3D | CD3E | CD3G | CD4 | CD40 | CD40LG | CD44 | CD45RA | CD45RB | CD45RO | CD47 |
| CD48 | CD5 | CD58 | CD6 | CD68 | CD69 | CD7 | CD70 | CD74 | CD79A | CD79B |
| CD80 | CD84 | CD86 | CD8A | CD8B | CD96 | CDC20 | CDC25C | CDH1 | CDH11 | CDH2 |
| CDH5 | CDK2 | CDK4 | CDK6 | CDKN1A | CDKN1C | CDKN2A | CDKN2B | CEACAM3 | CEBPB | CENPF |
| CEP55 | CES3 | CHUK | CLEC14A | CLEC4E | CLEC5A | CLEC7A | CLECL1 | CMKLR1 | CNTFR | COL11A1 |
| COL11A2 | COL17A1 | COL4A5 | COL5A1 | COL6A3 | COMP | CPA3 | CR1 | CR2 | CRABP2 | CRTAM |
| CSF1 | CSF1R | CSF2 | CSF2RB | CSF3 | CSF3R | CST2 | CTAG1B | CTLA4 | CTNNB1 | CTSS |
| CTSW | CX3CL1 | CX3CR1 | CXCL1 | CXCL10 | CXCL11 | CXCL12 | CXCL13 | CXCL14 | CXCL16 | CXCL2 |
| CXCL3 | CXCL5 | CXCL6 | CXCL8 | CXCL9 | CXCR2 | CXCR3 | CXCR4 | CXCR5 | CXCR6 | CXorf36 |
| CYBB | DAB2 | DDB2 | DEFB134 | DEPTOR | DKK1 | DLL1 | DLL4 | DNMT1 | DNMT3 | DPP4 |
| DTX3L | DTX4 | DUSP1 | DUSP2 | DUSP5 | E2F1 | E2F3 | EDN1 | EGF | EGFR | EGR1 |
| EGR2 | EIF2AK2 | EIF2B4 | EIF4EBP1 | EIF5AL1 | ELOB | ENO1 | ENTPD1 | EOMES | EPCAM | EPM2AIP1 |
| ERBB2 | ERO1A | ESR1 | EXO1 | EZH2 | F2RL1 | FADD | FAM124B | FAM30A | FANCA | FAP |
| FAS | FASLG | FBP1 | FCAR | FCGR1A | FCGR2A | FCGR2B | FCGR3A/B | FCGRT | FCN1 | FCRL2 |
| FGF13 | FGF18 | FGF9 | FGFR1 | FLNB | FLT1 | FOSL1 | FOXM1 | FOXP3 | FPR1 | FPR3 |
| FSTL3 | FUT4 | FYN | FZD8 | FZD9 | GAS1 | GATA3 | GBP1 | GBP2 | GBP4 | GHR |
| GIMAP4 | GIMAP6 | GLI1 | GLS | GLUD1 | GLUL | GMIP | GNG4 | GNLY | GOT1 | GOT2 |
| GPC4 | GPR160 | GPSM3 | GZMA | GZMB | GZMH | GZMK | GZMM | H2AFX | HAVCR2 | HCK |
| HDAC11 | HDAC3 | HDAC4 | HDAC5 | HDC | HELLS | HERC6 | HES1 | HEY1 | HIF1A | HK1 |
| HK2 | HLA-A | HLA-B | HLA-C | HLA-DMA | HLA-DMB | HLA-DOA | HLA-DOB | HLA-DPA1 | HLA-DPB1 | HLA-DQA1 |
| HLA-DQA2 | HLA-DQB1 | HLA-DRA | HLA-DRB1 | HLA-DRB5 | HLA-E | HLA-F | HMGA1 | HMGB1 | HNF1A | HRAS |
| HSD11B1 | ICAM1 | ICAM2 | ICAM3 | ICAM5 | ICOS | ICOSLG | ID4 | IDO1 | IER3 | IFI16 |
| IFI27 | IFI35 | IFI6 | IFIH1 | IFIT1 | IFIT2 | IFIT3 | IFITM1 | IFITM2 | IFNA1 | IFNAR1 |
| IFNG | IFNGR1 | IFNGR2 | IGF2R | IHH | IKBKB | IKBKG | IL10 | IL10RA | IL11 | IL11RA |
| IL12RB2 | IL15 | IL16 | IL17A | IL18 | IL18R1 | IL1A | IL1B | IL1R2 | IL1RN | IL2 |
| IL21 | IL21R | IL22RA1 | IL24 | IL2RA | IL2RB | IL2RG | IL32 | IL33 | IL34 | IL4 |
| IL6 | IL6R | IL7R | INHBA | IRF1 | IRF2 | IRF3 | IRF4 | IRF5 | IRF7 | IRF8 |
| IRF9 | ISG15 | ITGA1 | ITGA2 | ITGA4 | ITGA6 | ITGAE | ITGAL | ITGAM | ITGAV | ITGAX |
| ITGB2 | ITGB3 | ITGB8 | ITPK1 | JAG1 | JAG2 | JAK1 | JAK2 | JAK3 | KAT2B | KDR |
| KIF2C | KIR2DL3 | KIR3DL1 | KIR3DL2 | KIT | KLRB1 | KLRD1 | KLRK1 | KRAS | LAG3 | LAIR1 |
| LAMA1 | LAMB3 | LAMC2 | LCK | LDHA | LDHB | LGALS9 | LIF | LILRA1 | LILRA3 | LILRA5 |
| LILRB2 | LILRB4 | LMP1 | LOXL2 | LRRC32 | LTB | LTBP1 | LY9 | LY96 | LYZ | MAGEA1 |
| MAGEA12 | MAGEA3/A6 | MAGEA4 | MAGEB2 | MAGEC1 | MAGEC2 | MAML2 | MAP3K12 | MAP3K5 | MAP3K7 | MAP3K8 |
| MAPK10 | MARCO | MB21D1 | MELK | MET | MFGE8 | MFNG | MGMT | MIB1 | MICA | MICB |
| MKI67 | MLANA | MLH1 | MME | MMP1 | MMP7 | MMP9 | MMRN2 | MRC1 | MRE11 | MS4A1 |
| MS4A2 | MS4A4A | MS4A6A | MSH2 | MSH6 | MTOR | MUC1 | MX1 | MXI1 | MYC | MYCT1 |
| MYD88 | NANOG | NBN | NCAM1 | NCR1 | NDUFA4L2 | NECTIN1 | NECTIN2 | NEIL1 | NF1 | NFAM1 |
| NFATC2 | NFIL3 | NFKB1 | NFKB2 | NFKBIA | NFKBIE | NGFR | NID2 | NKG7 | NLRC5 | NLRP3 |
| NOD2 | NOS2 | NOTCH1 | NOTCH2 | NRAS | NT5E | OAS1 | OAS2 | OAS3 | OASL | OLFML2B |
| OLR1 | OTOA | P2RY13 | P4HA1 | P4HA2 | PALMD | PARP12 | PARP4 | PARP9 | PC | PCK2 |
| PDCD1 | PDCD1LG2 | PDGFA | PDGFB | PDGFRA | PDGFRB | PDK1 | PDZK1IP1 | PECAM1 | PF4 | PFKFB3 |
| PFKM | PGPEP1 | PIAS4 | PIK3CA | PIK3CD | PIK3CG | PIK3R1 | PIK3R2 | PIK3R5 | PIM1 | PIM2 |
| PKM | PLA1A | PLA2G2A | PLOD2 | PMS2 | PNOC | POLD1 | PPARG | PPARGC1B | PRDM1 | PRF1 |
| PRKAA2 | PRKACB | PRKCA | PRKX | PRLR | PROM1 | PRR5 | PSMB10 | PSMB5 | PSMB8 | PSMB9 |
| PTCD2 | PTEN | PTGER4 | PTGS2 | PTPN11 | PTPRC | PVR | PVRIG | RAD50 | RAD51 | RAD51C |
| RASAL1 | RASGRF1 | RB1 | RBL2 | RELA | RELB | RELN | REN | RICTOR | RIPK1 | RIPK2 |
| RIPK3 | RNLS | ROBO4 | ROCK1 | ROR2 | RORC | RPL23 | RPL7A | RPS6KB1 | RPTOR | RRM2 |
| RSAD2 | RUNX3 | S100A12 | S100A8 | S100A9 | SAMD9 | SAMSN1 | SBNO2 | SDC1 | SELE | SELL |
| SELP | SERPINA1 | SERPINB5 | SERPINH1 | SFRP1 | SFRP4 | SFXN1 | SGK1 | SH2D1A | SHC2 | SIGLEC1 |
| SIGLEC5 | SIGLEC8 | SIRPA | SIRPB2 | SLAMF7 | SLC11A1 | SLC16A1 | SLC1A5 | SLC2A1 | SLC7A5 | SMAD5 |
| SMAP1 | SNAI1 | SNCA | SOCS1 | SOX10 | SOX11 | SOX2 | SP1 | SPIB | SPP1 | SPRY4 |
| SREBF1 | SRP54 | STAT1 | STAT2 | STAT3 | STAT4 | STC1 | SYK | TAF3 | TAP1 | TAP2 |
| TAPBP | TAPBPL | TBX21 | TBXAS1 | TCF3 | TCL1A | TDO2 | TGFB1 | TGFB2 | TGFB3 | TGFBR1 |
| TGFBR2 | THBD | THBS1 | THY1 | TIA1 | TICAM1 | TIE1 | TIGIT | TLR1 | TLR2 | TLR3 |
| TLR4 | TLR5 | TLR7 | TLR8 | TLR9 | TMEM140 | TMEM173 | TNF | TNFAIP3 | TNFAIP6 | TNFRSF10B |
| TNFRSF10C | TNFRSF10D | TNFRSF11A | TNFRSF11B | TNFRSF14 | TNFRSF17 | TNFRSF18 | TNFRSF1A | TNFRSF1B | TNFRSF25 | TNFRSF4 |
| TNFRSF8 | TNFRSF9 | TNFSF10 | TNFSF12 | TNFSF13 | TNFSF13B | TNFSF18 | TNFSF4 | TNFSF8 | TNFSF9 | TNKS |
| TP53 | TPI1 | TPM1 | TPSAB1/B2 | TRAF1 | TRAT1 | TREM1 | TREM2 | TRIM21 | TSLP | TTC30A |
| TWF1 | TWIST1 | TWIST2 | TYMP | TYMS | UBA7 | UBE2C | UBE2T | ULBP2 | VCAM1 | VCAN |
| VEGFA | VEGFB | VEGFC | VHL | VSIR | VTCN1 | WDR76 | WNT10A | WNT11 | WNT2 | WNT2B |
| WNT3A | WNT4 | WNT5A | WNT5B | WNT7B | XCL1/2 | ZAP70 | ZC3H12A | ZEB1 | ZEB2 | ABCF1 |
| DNAJC14 | ERCC3 | G6PD | GUSB | MRPL19 | NRDE2 | OAZ1 | POLR2A | PSMC4 | PUM1 | SDHA |
| SF3A1 | STK11IP | TBC1D10B | TBP | TFRC | TLK2 | TMUB2 | UBB |  |  |  |

**Supplemental Table 2. Summary of sources for the selected and created GSs**. The final dataset contained 157 cell phenotypes and 1077 signaling pathways, distributed as follows:

| **SOURCE** | **LINK** | **NUMBER OF GS** |
| --- | --- | --- |
| GOBP | <https://www.gsea-msigdb.org/> | 480 |
| REACTOME | <https://www.gsea-msigdb.org/> | 178 |
| MANUAL | <https://pubmed.ncbi.nlm.nih.gov/> | 139 |
| GOCC | <https://www.gsea-msigdb.org/> | 69 |
| WIKIPATHWAYS | <https://www.gsea-msigdb.org/> | 63 |
| BIOCARTA | <https://www.gsea-msigdb.org/> | 61 |
| PID | https://www.gsea-msigdb.org/ | 55 |
| GENEONTOLOGY | <http://geneontology.org/> | 33 |
| FANTOM | <https://fantom.gsc.riken.jp/5/> | 17 |
| MSIGDB | <https://www.gsea-msigdb.org/> | 16 |
| GENEVESTIGATOR | <https://genevestigator.com/> | 16 |
| NOVERSHTERN | <https://doi.org/10.1016%2Fj.cell.2011.01.004> | 15 |
| BLUEPRINT | <http://blueprint-data.bsc.es/#!/> | 12 |
| SCTYPE | <https://github.com/IanevskiAleksandr/sc-type> | 12 |
| EUROPEPMC | <https://www.ebi.ac.uk/biostudies/europepmc/studies/S-EPMC6472943/> | 11 |
| KEGG | <https://www.gsea-msigdb.org/> | 11 |
| HALLMARK | <https://www.gsea-msigdb.org/> | 11 |
| HPCA | <https://www.humancellatlas.org/> | 9 |
| HPA | <https://www.proteinatlas.org/> | 7 |
| CELLXGENE | <https://cellxgene.cziscience.com/gene-expression/> | 6 |
| NABA | <https://www.gsea-msigdb.org/> | 5 |
| IRIS | <https://www.immgen.org/> | 4 |
| SIG | <https://www.gsea-msigdb.org/> | 2 |
| ENCODE | <https://www.encodeproject.org/> | 1 |
| SA | <https://www.gsea-msigdb.org/> | 1 |

**Supplemental Table 3. Summary of clinical data for all classic Hodgkin Lymphoma patients**

|  | | Total | Unfavorable | Favorable | p-value |
| --- | --- | --- | --- | --- | --- |
| Age | |  |  |  |  |
|  | ≥ 65 years | 4 (16%) | 1 (25%) | 3 (75%) | NS |
|  | < 65 years | 21 (84%) | 13 (62%) | 8 (38%) |  |
| Gender | |  |  |  |  |
|  | Male | 14 (56%) | 11 (79%) | 3 (21%) | 0.017 |
|  | Female  No data | 10 (40%)  1 (4%) | 3 (30%)  0 0(%) | 7 (70%)  1 (100%) |  |
| cHL subtype | |  |  |  |  |
|  | NS | 11 (44%) | 8 (73%) | 3 (27%) | NS |
|  | Others  No data | 12 (48%)  2 (8%) | 5 (42%)  1 (50%) | 7 (58%)  1 (50%) |  |
| Ann Arbor stage | |  |  |  |  |
|  | IV | 10 (40%) | 7 (70%) | 3 (30%) | NS |
|  | I-III | 15 (60%) | 7 (47%) | 8 (53%) |  |
| IPS | |  |  |  |  |
|  | ≥ 3 | 6 (24%) | 3 (50%) | 3 (50%) | NS |
|  | < 3  No data | 14 (56%)  5 (20%) | 9 (64%)  2 (40%) | 4 (36%)  3 (60%) |  |
| Epstein–Barr virus infection | |  |  |  |  |
|  | Positive | 6 (24%) | 3 (50%) | 3 (50%) | NS |
|  | Negative | 17 (68%) | 10 (59%) | 7 (41%) |  |
|  | No Data | 2 (8%) | 1 (50%) | 1 (50%) |  |
| Response to first treatment | |  |  |  |  |
|  | CR | 13 (52%) | 2 (15%) | 11 (85%) | <0.001 |
|  | PR + PD | 12 (48%) | 12 (100%) | 0 (0%) |  |

NS = nodular sclerosis; IPS = International Prognostic Score; CR = complete remission; PD = progressive disease; PR = partial response; Favorable = PFS > 2 years; Unfavorable = PFS < 2 years.

**Supplemental Table 4.** **Detailed results about inflammatory balance and MHC molecule distribution.** Components appearing in Figure 2 are highlighted in bold.

| **Variable** | **Source** | **Available plots** | **Variable enriched in:** | **p** |
| --- | --- | --- | --- | --- |
| **B2M GENE** | **Gene Expression** | **KM dichotomized 25%** | **U** | **0.049** |
|  |  | **KM dichotomized 33%** |  | **0.037** |
|  |  | **KM dichotomized 50%** |  | **0.025** |
| IFIT3 GENE | Gene Expression | KM dichotomized 50% | U | 0.030 |
|  |  | KM dichotomized 67% |  | 0.012 |
|  |  | KM dichotomized 75% |  | 0.054 |
|  |  | KM quartiles |  | 0.059 |
|  |  | KM tertiles |  | 0.077 |
| IFN ANTIVIRAL PATHWAY | REACTOME | Cox | U | 0.032 |
|  |  | KM dichotomized 25% |  | 0.013 |
|  |  | KM dichotomized 33% |  | 0.071 |
|  |  | KM dichotomized 50% |  | 0.043 |
|  |  | KM dichotomized 67% |  | 0.067 |
|  |  | KM quartiles |  | 0.051 |
|  |  | KM tertiles |  | 0.069 |
| IFNGR1 GENE | Gene Expression | KM quartiles | U | 0.069 |
|  |  | KM tertiles |  | 0.047 |
| **IFN-γ PATHWAY** | **Manual** | **Cox** | **U** | **0.048** |
|  |  | **KM dichotomized 25%** |  | **0.060** |
|  | **Manual + PID** | **KM dichotomized 50%** |  | **0.038** |
|  | **Manual** | **KM dichotomized 67%** |  | **0.090** |
|  | **Manual + PID** | **KM quartiles** |  | **0.033** |
| IFN-γ SIGNATURE | Manual | KM dichotomized 67% | U | 0.051 |
| **IL6 PATHWAY** | **BIOCARTA** | **Boxplot (t test)** | **U** | **0.099** |
|  | **Manual + PID** | **KM dichotomized 25%** |  | **0.010** |
|  | **BIOCARTA** | **KM dichotomized 50%** |  | **0.046** |
|  |  | **KM dichotomized 75%** |  | **0.067** |
| **MHC-I PATHWAY** | **Manual** | **Boxplot (Kruskal–Wallis)** | **RELAPSE** | **0.022** |
|  | **REACTOME** | **KM dichotomized 25%** | **U** | **0.022** |
| **MHC-II PATHWAY** | **REACTOME** | **Boxplot (t test)** | **NO_RELAPSE** | **0.052** |
| TGFB2 GENE | Gene Expression | Cox | F | 0.057 |
|  |  | KM dichotomized 25% |  | 0.059 |
| TGFBR1 GENE | Gene Expression | KM dichotomized 33% | F | 0.065 |
| **TGF-β PATHWAY** | **REACTOME + PID** | **Cox** | **F** | **0.012** |
|  | **PID** | **KM dichotomized 50%** |  | **0.082** |
|  | **PID + REACTOME** | **KM dichotomized 75%** |  | **0.003** |
| **TNF PATHWAY** | **BIOCARTA** | **KM dichotomized 25%** | **U** | **0.026** |
|  | **Manual** | **KM dichotomized 33%** |  | **0.081** |
|  |  | **KM dichotomized 75%** |  | **0.098** |

U = Unfavorable; F = Favorable; KM = Kaplan–Meier plot.

**Supplemental Table 5.** **Detailed results about myeloid cells.** Components appearing in Figure 3 are highlighted in bold.

| **Variable** | **Source** | **Available plots** | **Variable enriched in:** | **p** |
| --- | --- | --- | --- | --- |
| **ACTIVATED DENDRITIC CELLS** | **IRIS** | **KM dichotomized 25%** | **U** | **0.053** |
|  |  | **KM dichotomized 67%** |  | **0.033** |
|  |  | **KM tertiles** |  | **0.081** |
| ACTIVATED MACROPHAGES | Manual | KM dichotomized 25% | U | 0.046 |
| ACTIVATED MONOCYTES | CIBERSORTx | Boxplot (Kruskal–Wallis) | RELAPSE | 0.026 |
|  |  | KM dichotomized 33% | U | 0.042 |
|  |  | KM dichotomized 75% |  | 0.052 |
| ACTIVATED PLASMACYTOID DENDRITIC CELLS | CIBERSORTx | KM dichotomized 25% | U | 0.050 |
| BASOPHILS | NOVERSHTERN | Cox | U | 0.034 |
|  |  | KM dichotomized 25% |  | 0.092 |
|  |  | KM dichotomized 50% |  | 0.100 |
|  |  | KM dichotomized 67% |  | 0.028 |
|  | NOVERSHTERN + Manual | KM tertiles |  | 0.078 |
| C1QA GENE | Gene Expression | Boxplot (Kruskal–Wallis) | RELAPSE | 0.036 |
| C1QB GENE | Gene Expression | KM dichotomized 67% | U | 0.019 |
|  |  | KM dichotomized 75% |  | 0.047 |
| CD14 GENE | Gene Expression | KM dichotomized 75% | U | 0.055 |
|  |  | KM tertiles |  | 0.014 |
| **CD14+ CELLS** | **Manual** | **Boxplot (t test)** | **RELAPSE** | **0.030** |
| CD1C GENE | Gene Expression | Boxplot (t test) | U | 0.038 |
| CD23 GENE (MARKER OF fDCs) | Gene Expression | Boxplot (Kruskal–Wallis) | NO RELAPSE | 0.011 |
| **CREB PATHWAY** | **BIOCARTA** | **Boxplot (t test)** | **U** | **0.078** |
|  |  | **Cox** |  | **0.017** |
|  |  | **KM dichotomized 25%** |  | **0.016** |
|  |  | **KM dichotomized 33%** |  | **0.012** |
|  |  | **KM dichotomized 50%** |  | **0.038** |
| **DAP12 PATHWAY** | **REACTOME + Manual** | **Boxplot (t test)** | **U** | **0.008** |
|  |  | **Cox** |  | **0.005** |
|  | **REACTOME** | **KM dichotomized 25%** |  | **0.046** |
|  |  | **KM dichotomized 33%** |  | **0.014** |
|  |  | **KM dichotomized 50%** |  | **0.001** |
|  | **REACTOME + Manual** | **KM dichotomized 67%** |  | **0.024** |
|  | **REACTOME** | **KM dichotomized 75%** |  | **0.033** |
|  | **Manual + REACTOME** | **KM quartiles** |  | **0.003** |
|  | **REACTOME** | **KM tertiles** |  | **0.050** |
| EOSINOPHILS | HPA | Boxplot (t test) | RELAPSE | 0.014 |
|  | Manual | KM dichotomized 75% | U | 0.098 |
|  |  | KM tertiles |  | 0.010 |
| **FCGR PATHWAY** | **REACTOME** | **Boxplot (t test)** | **RELAPSE** | **0.073** |
|  |  | **KM dichotomized 25%** | **U** | **0.050** |
|  |  | **KM dichotomized 50%** |  | **0.048** |
|  |  | **KM quartiles** |  | **0.046** |
| FCGR1A GENE | Gene Expression | Boxplot (Kruskal–Wallis) | RELAPSE | 0.086 |
| GRANULOCYTES | Manual | Boxplot (t test) | RELAPSE | 0.095 |
|  |  | KM dichotomized 33% | U | 0.062 |
|  |  | KM dichotomized 75% |  | 0.055 |
|  |  | KM tertiles |  | 0.051 |
| **IL10 PATHWAY** | **REACTOME** | **Boxplot (t test)** | **RELAPSE** | **0.069** |
|  |  | **KM dichotomized 25%** | **U** | **0.060** |
|  |  | **KM dichotomized 33%** |  | **0.063** |
|  |  | **KM dichotomized 75%** |  | **0.032** |
| **IL12 PATHWAY** | **PID + BIOCARTA + Manual** | **KM dichotomized 67%** | **U** | **0.030** |
|  | **Manual + BIOCARTA + PID** | **KM dichotomized 75%** |  | **0.045** |
|  | **PID** | **KM quartiles** |  | **0.022** |
|  | **BIOCARTA + pid** | **KM tertiles** |  | **0.028** |
| IL23 PATHWAY | Manual | Cox | U | 0.058 |
|  |  | KM dichotomized 33% |  | 0.086 |
|  |  | KM dichotomized 50% |  | 0.021 |
|  |  | KM dichotomized 67% |  | 0.060 |
| **IL3 AND GMCSF PATHWAY** | **REACTOME** | **Cox** | **U** | **0.012** |
|  |  | **KM dichotomized 25%** |  | **0.022** |
|  |  | **KM dichotomized 33%** |  | **0.014** |
|  |  | **KM quartiles** |  | **0.026** |
|  |  | **KM tertiles** |  | **0.036** |
| **IMMATURE MACROPHAGES** | **Manual** | **Boxplot (t test)** | **F** | **0.053** |
|  |  | **KM dichotomized 67%** |  | **0.022** |
|  |  | **KM tertiles** |  | **0.024** |
| ISG15 GENE | Gene Expression | KM dichotomized 33% | U | 0.098 |
|  |  | KM dichotomized 50% |  | 0.024 |
|  |  | KM dichotomized 67% |  | 0.012 |
|  |  | KM dichotomized 75% |  | 0.054 |
|  |  | KM tertiles |  | 0.097 |
| **LYZ GENE** | **Gene Expression** | **Boxplot (Kruskal–Wallis)** | **RELAPSE** | **0.008** |
|  |  | **KM dichotomized 50%** | **U** | **0.079** |
|  |  | **KM dichotomized 67%** |  | **0.029** |
|  |  | **KM dichotomized 75%** |  | **0.073** |
|  |  | **KM quartiles** |  | **0.077** |
| **M1 MACROPHAGES** | **FANTOM + BLUEPRINT + Manual** | **Boxplot (t test)** | **RELAPSE** | **0.019** |
|  | **Manual** | **Cox** | **U** | **0.059** |
|  |  | **KM dichotomized 25%** |  | **0.065** |
|  |  | **KM dichotomized 33%** |  | **0.066** |
|  |  | **KM dichotomized 50%** |  | **0.084** |
| **M2 MACROPHAGES** | **Manual** | **Boxplot (Kruskal–Wallis)** | **U** | **0.071** |
|  |  | **Cox** |  | **0.071** |
|  |  | **KM dichotomized 25%** |  | **0.020** |
|  |  | **KM dichotomized 67%** |  | **0.080** |
| MACROPHAGE PATHWAY | WIKIPATHWAYS | Boxplot (t test) | RELAPSE | 0.007 |
| **MACROPHAGES** | **Manual + BLUEPRINT + HPCA + FANTOM** | **Boxplot (t test)** | **RELAPSE** | **0.009** |
|  | **FANTOM** | **KM dichotomized 25%** | **U** | **0.099** |
|  |  | **KM dichotomized 33%** |  | **0.078** |
| **MIF PATHWAY** | **Manual** | **Boxplot (t test)** | **F** | **0.045** |
|  |  | **Cox** |  | **0.046** |
|  |  | **KM dichotomized 75%** |  | **0.001** |
| **MONOCYTES** | **Manual + CIBERSORTx + FANTOM + NOVERSHTERN + HPA + BLUEPRINT** | **Boxplot (t test)** | **RELAPSE** | **0.008** |
|  | **CIBERSORTx** | **KM dichotomized 25%** | **U** | **0.059** |
|  | **Manual + IRIS + NOVERSHTERN** | **KM dichotomized 50%** |  | **0.047** |
|  | **Manual + NOVERSHTERN + BLUEPRINT + IRIS** | **KM dichotomized 67%** |  | **0.019** |
|  | **BLUEPRINT** | **KM dichotomized 75%** |  | **0.050** |
|  | **NOVERSHTERN + BLUEPRINT + IRIS** | **KM quartiles** |  | **0.006** |
| MYELOID CELLS | Manual | Boxplot (t test) | RELAPSE | 0.047 |
| **MYELOID DENDRITIC CELLS** | **Manual + CIBERSORTx** | **Boxplot (t test)** | **RELAPSE** | **0.025** |
|  | **Manual** | **Boxplot (Kruskal–Wallis)** | **U** | **0.071** |
|  |  | **KM dichotomized 25%** |  | **0.017** |
|  | **NOVERSHTERN + Manual** | **KM dichotomized 33%** |  | **0.074** |
|  | **Manual** | **KM dichotomized 50%** |  | **0.070** |
| **MYELOID-DERIVED SUPPRESSOR CELLS (MDSC)** | **Manual** | **Boxplot (t test)** | **RELAPSE** | **0.065** |
|  | **BLUEPRINT** |  | **U** | **0.012** |
|  |  | **Cox** |  | **0.079** |
|  |  | **KM dichotomized 25%** |  | **0.001** |
|  | **BLUEPRINT + Manual** | **KM dichotomized 33%** |  | **0.015** |
|  | **BLUEPRINT** | **KM dichotomized 50%** |  | **0.083** |
|  |  | **KM quartiles** |  | **0.046** |
|  |  | **KM tertiles** |  | **0.065** |
| NEUTROPHIL DEGRANULATION PATHWAY | Manual + REACTOME | Boxplot (t test) | RELAPSE | 0.022 |
|  | Manual | KM dichotomized 67% | U | 0.052 |
|  |  | KM quartiles |  | 0.096 |
| NEUTROPHILS | GENEVESTIGATOR | Boxplot (t test) | RELAPSE | 0.080 |
|  |  | KM dichotomized 67% | U | 0.019 |
|  |  | KM dichotomized 75% |  | 0.032 |
|  | Manual | KM quartiles |  | 0.087 |
|  |  | KM tertiles |  | 0.001 |
| **NFAT PATHWAY** | **Manual** | **Boxplot (t test)** | **NO RELAPSE** | **0.006** |
| **NON-CLASSICAL MONOCYTES** | **HPA** | **Boxplot (t test)** | **RELAPSE** | **0.016** |
|  | **Manual** | **Cox** | **U** | **0.035** |
|  | **CIBERSORTx** | **KM dichotomized 67%** |  | **0.052** |
|  | **Manual** | **KM dichotomized 75%** |  | **0.026** |
|  | **CIBERSORTx** | **KM tertiles** |  | **0.068** |
| PHAGOCYTOSIS PATHWAY | REACTOME | Boxplot (t test) | RELAPSE | 0.043 |
|  |  | Cox | U | 0.037 |
|  |  | KM dichotomized 25% |  | 0.050 |
|  |  | KM dichotomized 33% |  | 0.025 |
|  |  | KM dichotomized 50% |  | 0.008 |
|  |  | KM dichotomized 67% |  | 0.026 |
|  |  | KM tertiles |  | 0.079 |
| **PLASMACYTOID DENDRITIC CELLS** | **CIBERSORTx + Manual** | **Boxplot (t test)** | **RELAPSE** | **0.032** |
|  | **FANTOM + Manual** | **Cox** | **U** | **0.046** |
|  | **Manual** | **KM dichotomized 25%** |  | **0.072** |
|  | **FANTOM + Manual** | **KM dichotomized 50%** |  | **0.045** |
|  | **Manual** | **KM dichotomized 67%** |  | **0.021** |
|  | **HPA + Manual** | **KM quartiles** |  | **0.021** |
| PTPRC GENE | GENEVESTIGATOR | Cox | U | 0.010 |
|  |  | KM dichotomized 25% |  | 0.041 |
|  |  | KM dichotomized 33% |  | 0.008 |
|  |  | KM dichotomized 50% |  | 0.006 |
|  |  | KM dichotomized 67% |  | 0.010 |
|  |  | KM quartiles |  | 0.002 |
|  |  | KM tertiles |  | 0.012 |
| **RESTING MONOCYTES** | **CIBERSORTx** | **Boxplot (Kruskal–Wallis)** | **RELAPSE** | **0.033** |
| SPIB GENE | GENEVESTIGATOR | Cox | U | 0.098 |
|  |  | KM dichotomized 25% |  | 0.060 |
|  |  | KM dichotomized 33% |  | 0.011 |
|  |  | KM dichotomized 50% |  | 0.016 |
| TRIM21 GENE | GENEVESTIGATOR | KM dichotomized 25% | U | 0.093 |
|  |  | KM dichotomized 33% |  | 0.076 |
|  |  | KM dichotomized 50% |  | 0.041 |
|  |  | KM dichotomized 67% |  | 0.001 |
|  |  | KM dichotomized 75% |  | 0.034 |
|  |  | KM quartiles |  | 0.061 |
|  |  | KM tertiles |  | 0.012 |

U = Unfavorable; F = Favorable; KM = Kaplan–Meier plot.

**Supplemental Table 6.** **Detailed results about cytotoxic cells.** Components appearing in Figure 4 are highlighted in bold.

| **Variable** | **Source** | **Available plots** | **Variable enriched in:** | **p** |
| --- | --- | --- | --- | --- |
| **ACTIVATED MATURE NK CELLS** | **CIBERSORTx** | **Boxplot (t test)** | **F** | **0.084** |
|  |  | **KM dichotomized 50%** |  | **0.027** |
| CD244 GENE | Gene Expression | KM dichotomized 75% | U | 0.041 |
| CD48 GENE | Gene Expression | Cox | U | 0.029 |
|  |  | KM dichotomized 25% |  | 0.006 |
|  |  | KM dichotomized 67% |  | 0.015 |
|  |  | KM quartiles |  | 0.086 |
|  |  | KM tertiles |  | 0.018 |
| **CD8+ T CELL PATHWAY** | **BIOCARTA** | **KM dichotomized 25%** | **U** | **0.038** |
| **CD8+ T CELLS** | **CIBERSORTx** | **Cox** | **U** | **0.037** |
|  | **CIBERSORTx + FANTOM + HPCA** | **KM dichotomized 25%** |  | **0.001** |
|  | **HPCA + CIBERSORTx + FANTOM** | **KM dichotomized 33%** |  | **0.033** |
|  | **CIBERSORTx** | **KM dichotomized 50%** |  | **0.009** |
|  | **Manual** | **KM dichotomized 67%** |  | **0.097** |
|  | **CIBERSORTx** | **KM quartiles** |  | **0.054** |
|  | **Manual** | **KM tertiles** |  | **0.073** |
| **CENTRAL MEMORY CD8+ T CELLS** | **HPCA** | **KM dichotomized 25%** | **U** | **0.005** |
|  | **Manual** | **KM dichotomized 67%** |  | **0.040** |
| **EFFECTOR MEMORY CD8+ T CELLS** | **CIBERSORTx** | **Boxplot (Kruskal–Wallis)** | **U** | **0.008** |
|  | **CIBERSORTx** | **Cox** |  | **0.016** |
|  | **CIBERSORTx + HPCA + NOVERSHTERN** | **KM dichotomized 25%** |  | **0.003** |
|  | **CIBERSORTx + NOVERSHTERN** | **KM dichotomized 33%** |  | **0.064** |
|  | **CIBERSORTx** | **KM dichotomized 50%** |  | **0.027** |
|  | **CIBERSORTx** | **KM dichotomized 67%** |  | **0.014** |
|  | **CIBERSORTx + GENEVESTIGATOR** | **KM dichotomized 75%** |  | **0.017** |
|  | **CIBERSORTx** | **KM quartiles** |  | **0.094** |
|  | **CIBERSORTx** | **KM tertiles** |  | **0.096** |
| GNLY GENE | Gene Expression | Boxplot (t test) | U | 0.067 |
|  |  | Cox |  | 0.045 |
|  |  | KM dichotomized 25% |  | 0.004 |
|  |  | KM dichotomized 33% |  | 0.008 |
|  |  | KM dichotomized 67% |  | 0.051 |
|  |  | KM quartiles |  | 0.022 |
|  |  | KM tertiles |  | 0.002 |
| **IMMATURE NK CELLS** | **CIBERSORTx** | **Boxplot (t test)** | **RELAPSE** | **0.009** |
|  |  | **KM dichotomized 67%** | **U** | **0.026** |
|  |  | **KM dichotomized 75%** |  | **0.060** |
| **KIR3DL1 GENE** | **Gene Expression** | **Boxplot (Kruskal–Wallis)** | **NO_RELAPSE** | **0.019** |
| KIR3DL2 GENE | Gene Expression | KM quartiles | F | 0.022 |
| **KLRB1 GENE** | **Gene Expression** | **KM dichotomized 25%** | **F** | **0.049** |
|  |  | **KM dichotomized 33%** |  | **0.077** |
|  |  | **KM dichotomized 67%** |  | **0.054** |
|  |  | **KM tertiles** |  | **0.082** |
| **MATURE NK CELLS** | **CIBERSORTx** | **Cox** | **F** | **0.088** |
|  |  | **KM dichotomized 67%** |  | **0.008** |
|  |  | **KM dichotomized 75%** |  | **0.072** |
|  |  | **KM quartiles** |  | **0.092** |
| **NAIVE CD8+ T CELLS** | **CIBERSORTx** | **Boxplot (t test)** | **RELAPSE** | **0.036** |
|  | **CIBERSORTx** | **Boxplot (t test)** | **U** | **0.054** |
|  | **CIBERSORTx** | **Cox** |  | **0.031** |
|  | **Manual** | **KM dichotomized 25%** |  | **0.019** |
|  | **CIBERSORTx** | **KM dichotomized 67%** |  | **0.009** |
| GZMA GENE | Gene Expression | KM dichotomized 25% | U | 0.092 |
|  |  | KM dichotomized 33% |  | 0.052 |
| **GZMB GENE** | **Gene Expression** | **KM dichotomized 67%** | **U** | **0.024** |
|  |  | **KM dichotomized 75%** |  | **0.048** |
| GZMH GENE | Gene Expression | KM dichotomized 33% | U | 0.094 |
|  |  | KM dichotomized 75% |  | 0.047 |
| **IMMATURE CD8+ T CELLS** | **Manual** | **KM dichotomized 50%** | **F** | **0.028** |
| TEMRA CD8+ T CELLS | CIBERSORTx | Boxplot (Kruskal–Wallis) | U | 0.095 |
|  |  | Cox |  | 0.087 |
|  |  | KM dichotomized 25% |  | 0.010 |
| **TRKA PATHWAY** | **BIOCARTA** | **Cox** | **U** | **0.057** |
|  |  | **KM dichotomized 25%** |  | **0.013** |
|  |  | **KM dichotomized 33%** |  | **0.016** |
|  |  | **KM dichotomized 50%** |  | **0.007** |
|  |  | **KM dichotomized 67%** |  | **0.086** |
|  | **SA + BIOCARTA** | **KM quartiles** |  | **0.002** |
|  | **BIOCARTA** | **KM tertiles** |  | **0.033** |

U = Unfavorable; F = Favorable; KM = Kaplan–Meier plot.

**Supplemental Table 7.** **Detailed results about stromal components.** Components appearing in Figure 5 are highlighted in bold.

| **Variable** | **Source** | **Available plots** | **Variable enriched in:** | **p** |
| --- | --- | --- | --- | --- |
| **ACTIN CYTOSKELETON PATHWAY** | **Manual** | **Boxplot (t test)** | **U** | **0.048** |
|  |  | **KM dichotomized 50%** |  | **0.056** |
|  | **KEGG + Manual + WIKIPATHWAYS** | **KM dichotomized 67%** |  | **0.008** |
|  | **WIKIPATHWAYS** | **KM dichotomized 75%** |  | **0.017** |
|  | **KEGG + WIKIPATHWAYS** | **KM quartiles** |  | **0.007** |
|  | **KEGG + Manual** | **KM tertiles** |  | **0.006** |
| **ANGIOGENESIS PATHWAY** | **PID** | **KM dichotomized 25%** | **U** | **0.053** |
|  |  | **KM dichotomized 33%** |  | **0.021** |
| ARNT2 GENE | Gene Expression | Boxplot (Kruskal–Wallis) | RELAPSE | 0.008 |
| CD31+ CELLS | Manual | KM dichotomized 50% | U | 0.046 |
| CDH2 GENE | Gene Expression | Boxplot (Kruskal–Wallis) | U | 0.037 |
|  |  | KM dichotomized 67% |  | 0.038 |
| CTNNB1 GENE | Gene Expression | KM dichotomized 67% | U | 0.062 |
|  |  | KM dichotomized 75% |  | 0.024 |
|  |  | KM quartiles |  | 0.007 |
| D4GDI PATHWAY | BIOCARTA | Cox | U | 0.056 |
|  |  | KM dichotomized 25% |  | 0.049 |
|  |  | KM dichotomized 33% |  | 0.041 |
| DPP4 GENE | Gene Expression | Boxplot (t test) | U | 0.019 |
|  |  | Cox |  | 0.043 |
|  |  | KM dichotomized 25% |  | 0.023 |
|  |  | KM tertiles |  | 0.041 |
| **FCERI PATHWAY** | **BIOCARTA** | **Cox** | **U** | **0.060** |
|  | **BIOCARTA + REACTOME + KEGG** | **KM dichotomized 25%** |  | **0.001** |
|  | **REACTOME + KEGG** | **KM dichotomized 33%** |  | **0.014** |
|  | **PID** | **KM dichotomized 50%** |  | **0.086** |
|  |  | **KM dichotomized 75%** |  | **0.098** |
|  |  | **KM quartiles** |  | **0.007** |
| **FGF PATHWAY** | **PID** | **Boxplot (t test)** | **U** | **0.042** |
| FGFR PATHWAY | REACTOME | Boxplot (Kruskal–Wallis) | U | 0.063 |
|  |  | KM dichotomized 25% |  | 0.009 |
|  |  | KM dichotomized 67% |  | 0.095 |
|  |  | KM dichotomized 75% |  | 0.050 |
|  |  | KM tertiles |  | 0.082 |
| GBP1 GENE | Gene Expression | KM dichotomized 25% | U | 0.060 |
|  |  | KM dichotomized 33% |  | 0.044 |
|  |  | KM dichotomized 50% |  | 0.001 |
|  |  | KM dichotomized 67% |  | 0.002 |
|  |  | KM dichotomized 75% |  | 0.043 |
|  |  | KM quartiles |  | 0.019 |
|  |  | KM tertiles |  | 0.012 |
| **HYPOXIA PATHWAY** | **REACTOME** | **Cox** | **U** | **0.067** |
|  |  | **KM dichotomized 75%** |  | **0.027** |
|  |  | **KM quartiles** |  | **0.001** |
| **IL33 GENE** | **Gene Expression** | **Boxplot (Kruskal–Wallis)** | **U** | **0.055** |
|  |  | **KM dichotomized 67%** |  | **0.045** |
|  |  | **KM dichotomized 75%** |  | **0.019** |
|  |  | **KM quartiles** |  | **0.014** |
|  |  | **KM tertiles** |  | **0.004** |
| **IL7 PATHWAY** | **WIKIPATHWAYS** | **KM dichotomized 25%** | **U** | **0.050** |
| **INTEGRINS PATHWAY** | **WIKIPATHWAYS + PID** | **Boxplot (t test)** | **U** | **0.051** |
|  | **PID** | **Cox** |  | **0.097** |
|  | **PID + WIKIPATHWAYS** | **KM dichotomized 33%** |  | **0.036** |
|  | **WIKIPATHWAYS** | **KM dichotomized 50%** |  | **0.056** |
|  | **Manual** | **KM dichotomized 67%** |  | **0.078** |
|  |  | **KM dichotomized 75%** |  | **0.030** |
|  |  | **KM quartiles** |  | **0.022** |
| LIF GENE | Gene Expression | Boxplot (Kruskal–Wallis) | U | 0.055 |
|  |  | KM dichotomized 67% |  | 0.003 |
|  |  | KM dichotomized 75% |  | 0.067 |
|  |  | KM quartiles |  | 0.081 |
|  |  | KM tertiles |  | 0.001 |
| **MAST CELLS** | **Manual** | **Boxplot (t test)** | **RELAPSE** | **0.012** |
|  | **Manual + FANTOM** | **Boxplot (t test)** | **U** | **0.039** |
|  | **Manual** | **Cox** |  | **0.009** |
|  |  | **KM dichotomized 25%** |  | **0.061** |
|  |  | **KM dichotomized 33%** |  | **0.048** |
|  | **CIBERSORTx + Manual** | **KM dichotomized 50%** |  | **0.041** |
|  |  | **KM dichotomized 67%** |  | **0.075** |
|  | **Manual** | **KM dichotomized 75%** |  | **0.028** |
|  | **FANTOM + CIBERSORTx** | **KM quartiles** |  | **0.013** |
|  | **Manual** | **KM tertiles** |  | **0.089** |
| **MEGAKARYOCYTE PROGENITORS** | **Manual** | **Cox** | **U** | **0.071** |
|  |  | **KM dichotomized 33%** |  | **0.021** |
|  |  | **KM dichotomized 67%** |  | **0.072** |
|  |  | **KM dichotomized 75%** |  | **0.033** |
|  |  | **KM tertiles** |  | **0.093** |
| **PDGF PATHWAY** | **Manual** | **Boxplot (t test)** | **U** | **0.049** |
|  | **WIKIPATHWAYS + Manual** | **Cox** |  | **0.064** |
|  |  | **KM dichotomized 25%** |  | **0.006** |
|  | **Manual + WIKIPATHWAYS** | **KM dichotomized 33%** |  | **0.031** |
|  | **Manual** | **KM dichotomized 67%** |  | **0.063** |
|  | **WIKIPATHWAYS** | **KM quartiles** |  | **0.049** |
|  | **WIKIPATHWAYS + Manual** | **KM tertiles** |  | **0.034** |
| PDGFRB PATHWAY | WIKIPATHWAYS | Boxplot (t test) | U | 0.073 |
|  | WIKIPATHWAYS + PID | Cox |  | 0.012 |
|  | WIKIPATHWAYS | KM dichotomized 25% |  | 0.008 |
|  | WIKIPATHWAYS + PID | KM dichotomized 33% |  | 0.000 |
|  | PID + WIKIPATHWAYS | KM dichotomized 67% |  | 0.075 |
|  | WIKIPATHWAYS | KM dichotomized 75% |  | 0.060 |
|  |  | KM quartiles |  | 0.089 |
|  | WIKIPATHWAYS + PID | KM tertiles |  | 0.002 |
| **PLATELET PATHWAY** | **Manual** | **KM dichotomized 33%** | **U** | **0.022** |
| PLATELETS | Manual | KM dichotomized 25% | U | 0.009 |
|  |  | KM dichotomized 50% |  | 0.097 |
| **PTP1B PATHWAY** | **PID** | **Boxplot (t test)** | **U** | **0.029** |
|  |  | **Cox** |  | **0.045** |
|  |  | **KM dichotomized 50%** |  | **0.087** |
|  |  | **KM dichotomized 67%** |  | **0.041** |
|  |  | **KM dichotomized 75%** |  | **0.042** |
|  |  | **KM tertiles** |  | **0.075** |
| **ROBO PATHWAY** | **REACTOME** | **Cox** | **U** | **0.035** |
|  |  | **KM dichotomized 33%** |  | **0.031** |
|  |  | **KM dichotomized 67%** |  | **0.035** |
| ROCK1 GENE | Gene Expression | Boxplot (Kruskal–Wallis) | U | 0.043 |
|  |  | Cox |  | 0.010 |
|  |  | KM dichotomized 25% |  | 0.045 |
|  |  | KM dichotomized 33% |  | 0.002 |
|  |  | KM dichotomized 50% |  | 0.003 |
|  |  | KM dichotomized 75% |  | 0.064 |
|  |  | KM quartiles |  | 0.004 |
|  |  | KM tertiles |  | 0.021 |
| SELP GENE | Gene Expression | KM dichotomized 25% | U | 0.050 |
|  |  | KM dichotomized 33% |  | 0.049 |
|  |  | KM tertiles |  | 0.089 |
| **SEMAPHORIN PATHWAY** | **REACTOME** | **Boxplot (t test)** | **U** | **0.006** |
|  |  | **Cox** |  | **0.006** |
|  |  | **KM dichotomized 25%** |  | **0.010** |
|  |  | **KM dichotomized 33%** |  | **0.006** |
|  |  | **KM dichotomized 50%** |  | **0.002** |
|  |  | **KM dichotomized 67%** |  | **0.031** |
|  |  | **KM quartiles** |  | **0.047** |
|  |  | **KM tertiles** |  | **0.007** |
| **SHP2 PATHWAY** | **PID** | **Boxplot (Kruskal–Wallis)** | **U** | **0.049** |
|  |  | **Cox** |  | **0.069** |
|  |  | **KM dichotomized 50%** |  | **0.039** |
|  |  | **KM dichotomized 67%** |  | **0.010** |
|  |  | **KM dichotomized 75%** |  | **0.003** |
|  |  | **KM quartiles** |  | **0.056** |
|  |  | **KM tertiles** |  | **0.011** |
| SPP1 GENE | Gene Expression | Boxplot (Kruskal–Wallis) | U | 0.055 |
|  |  | KM dichotomized 67% |  | 0.040 |
|  |  | KM dichotomized 75% |  | 0.096 |
| **TCPTP PATHWAY** | **PID** | **Boxplot (t test)** | **U** | **0.004** |
|  |  | **Cox** |  | **0.040** |
|  |  | **KM dichotomized 33%** |  | **0.046** |
|  |  | **KM dichotomized 50%** |  | **0.007** |
|  |  | **KM dichotomized 67%** |  | **0.029** |
|  |  | **KM quartiles** |  | **0.091** |
| TMUB2 GENE | Gene Expression | Boxplot (Kruskal–Wallis) | RELAPSE | 0.086 |
|  |  | Boxplot (Kruskal–Wallis) | U | 0.090 |
|  |  | Cox |  | 0.009 |
|  |  | KM dichotomized 50% |  | 0.004 |
|  |  | KM tertiles |  | 0.092 |
| **VEGF PATHWAY** | **BIOCARTA** | **Boxplot (t test)** | **U** | **0.010** |
|  | **Manual** | **KM dichotomized 67%** |  | **0.083** |
| VEGFR PATHWAY | PID | KM dichotomized 50% | U | 0.046 |
|  |  | KM quartiles |  | 0.003 |

U = Unfavorable; F = Favorable; KM = Kaplan–Meier plot.

**Supplemental Table 8.** **Detailed results about CD4+ T cells.** Components appearing in Figure 6 are highlighted in bold.

| **Variable** | **Source** | **Available plots** | **Variable enriched in:** | **p** |
| --- | --- | --- | --- | --- |
| **ACTIVATED EFFECTOR CD4+ T CELLS** | **CIBERSORTx** | **KM dichotomized 25%** | **F** | **0.090** |
|  |  | **KM dichotomized 33%** |  | **0.027** |
|  |  | **KM tertiles** |  | **0.035** |
|  |  | **Boxplot (Kruskal–Wallis)** | **NO RELAPSE** | **0.065** |
| **ACTIVATED EFFECTOR NAIVE CD4+ T CELLS** | **CIBERSORTx** | **KM dichotomized 25%** | **F** | **0.043** |
|  |  | **KM dichotomized 67%** |  | **0.061** |
|  |  | **KM dichotomized 75%** |  | **0.088** |
|  |  | **KM quartiles** |  | **0.031** |
|  |  | **KM tertiles** |  | **0.000** |
|  |  | **Boxplot (Kruskal–Wallis)** | **NO RELAPSE** | **0.086** |
| **ACTIVATED TH17 PRECURSORS** | **CIBERSORTx** | **KM dichotomized 50%** | **F** | **0.026** |
| **ACTIVATED TH2 PRECURSORS** | **CIBERSORTx** | **Boxplot (t test)** | **F** | **0.096** |
|  |  | **KM dichotomized 33%** |  | **0.047** |
|  |  | **KM dichotomized 50%** |  | **0.090** |
|  |  | **KM dichotomized 67%** |  | **0.010** |
|  |  | **KM dichotomized 75%** |  | **0.004** |
| **EFFECTOR MEMORY CD4+ T CELLS** | **CIBERSORTx** | **Cox** | **U** | **0.025** |
|  | **Manual** | **KM dichotomized 67%** |  | **0.080** |
|  | **Manual + CIBERSORTx** | **KM dichotomized 75%** |  | **0.027** |
|  | **BLUEPRINT + CIBERSORTx** | **KM quartiles** |  | **0.007** |
| **EFFECTOR NAIVE CD4+ T CELLS** | **CIBERSORTx** | **Cox** | **U** | **0.097** |
|  |  | **KM dichotomized 67%** |  | **0.068** |
|  |  | **KM dichotomized 75%** |  | **0.028** |
| **FOLLICULAR HELPER CD4+ T CELLS** | **CIBERSORTx** | **Cox** | **U** | **0.031** |
|  |  | **KM dichotomized 25%** |  | **0.008** |
|  |  | **KM dichotomized 33%** |  | **0.011** |
|  |  | **KM dichotomized 50%** |  | **0.034** |
|  | **CIBERSORTx + Manual** | **KM dichotomized 67%** |  | **0.001** |
|  | **CIBERSORTx** | **KM dichotomized 75%** |  | **0.002** |
|  | **Manual + CIBERSORTx** | **KM quartiles** |  | **0.021** |
|  | **CIBERSORTx** | **KM tertiles** |  | **0.005** |
| IL18R1 GENE | Gene Expression | KM dichotomized 33% | U | 0.076 |
|  |  | KM dichotomized 50% |  | 0.005 |
|  |  | KM dichotomized 67% |  | 0.077 |
|  |  | KM quartiles |  | 0.071 |
| **NAIVE CD4+ T CELLS** | **Manual** | **KM dichotomized 25%** | **U** | **0.003** |
|  |  | **KM dichotomized 33%** |  | **0.034** |
|  | **NOVERSHTERN** | **KM dichotomized 67%** |  | **0.080** |
|  | **NOVERSHTERN + Manual** | **KM quartiles** |  | **0.011** |
|  | **NOVERSHTERN** | **KM tertiles** |  | **0.078** |
| **REGULATORY NAIVE T CELLS** | **CIBERSORTx** | **Cox** | **U** | **0.042** |
| **REGULATORY T CELLS** | **CIBERSORTx** | **Cox** | **F** | **0.035** |
|  |  | **KM dichotomized 25%** |  | **0.060** |
|  |  | **KM dichotomized 33%** |  | **0.019** |
|  | **Manual + CIBERSORTx** | **KM tertiles** |  | **0.018** |
|  | **CIBERSORTx** | **Boxplot (t test)** | **NO RELAPSE** | **0.098** |
| **TH1 CELLS** | **CIBERSORTx** | **Boxplot (Kruskal–Wallis)** | **RELAPSE** | **0.013** |
|  |  | **Boxplot (t test)** | **U** | **0.054** |
|  |  | **Cox** |  | **0.046** |
|  |  | **KM dichotomized 25%** |  | **0.001** |
|  |  | **KM dichotomized 33%** |  | **0.000** |
|  | **Manual** | **KM dichotomized 67%** |  | **0.079** |
|  | **CIBERSORTx** | **KM quartiles** |  | **0.000** |
| **TH1 PRECURSORS** | **CIBERSORTx** | **Boxplot (Kruskal–Wallis)** | **U** | **0.093** |
|  |  | **Cox** |  | **0.019** |
|  |  | **KM dichotomized 25%** |  | **0.013** |
|  |  | **KM dichotomized 33%** |  | **0.041** |
|  |  | **KM dichotomized 50%** |  | **0.063** |
|  |  | **KM dichotomized 67%** |  | **0.046** |
|  |  | **KM dichotomized 75%** |  | **0.005** |
|  |  | **KM quartiles** |  | **0.058** |
|  |  | **KM tertiles** |  | **0.026** |
| **TH17 CELLS** | **Manual** | **Boxplot (t test)** | **RELAPSE** | **0.045** |
|  | **CIBERSORTx** |  | **U** | **0.022** |
|  |  | **Cox** |  | **0.045** |
|  |  | **KM dichotomized 67%** |  | **0.064** |
|  |  | **KM dichotomized 75%** |  | **0.088** |
| TH17 DIFFERENTIATION PATHWAY | Manual | KM dichotomized 67% | U | 0.051 |
| **TH17 PRECURSORS** | **CIBERSORTx** | **Boxplot (Kruskal–Wallis)** | **F** | **0.042** |
| **TH2 CELLS** | **Manual** | **Cox** | **U** | **0.087** |
|  | **Manual + CIBERSORTx** | **KM dichotomized 25%** |  | **0.033** |
|  |  | **KM dichotomized 33%** |  | **0.040** |
|  | **Manual** | **KM dichotomized 67%** |  | **0.083** |
|  |  | **KM dichotomized 75%** |  | **0.018** |
| **TR1 CELLS** | **CIBERSORTx** | **KM dichotomized 25%** | **U** | **0.005** |
|  |  | **KM dichotomized 33%** |  | **0.013** |
|  |  | **KM tertiles** |  | **0.070** |
| **TR17 CELLS** | **CIBERSORTx** | **Boxplot (t test)** | **F** | **0.050** |
|  |  | **Cox** |  | **0.006** |
|  |  | **KM dichotomized 25%** |  | **0.006** |
|  |  | **KM dichotomized 33%** |  | **0.014** |
|  |  | **KM dichotomized 50%** |  | **0.030** |
|  |  | **KM dichotomized 67%** |  | **0.036** |
|  |  | **KM dichotomized 75%** |  | **0.053** |
|  |  | **KM quartiles** |  | **0.014** |
|  |  | **KM tertiles** |  | **0.032** |
|  |  | **Boxplot (t test)** | **NO RELAPSE** | **0.054** |
| **TR22 CELLS** | **CIBERSORTx** | **Boxplot (t test)** | **U** | **0.043** |
|  |  | **Cox** |  | **0.019** |
|  |  | **KM dichotomized 25%** |  | **0.054** |
|  |  | **KM dichotomized 50%** |  | **0.079** |
|  |  | **KM dichotomized 67%** |  | **0.020** |
|  |  | **KM dichotomized 75%** |  | **0.079** |
|  |  | **KM quartiles** |  | **0.079** |
|  |  | **KM tertiles** |  | **0.011** |

U = Unfavorable; F = Favorable; KM = Kaplan–Meier plot.

**Supplemental Table 9. Detailed clinical information and follow up.**

| **Case #** | **cHL subtype** | **Ann Arbor stage** | **Epstein–Barr virus** | **U/F classification** | **Age** | **Sex** | **IPS** | **First-line therapy** | **Response** | **Relapse** |
| --- | --- | --- | --- | --- | --- | --- | --- | --- | --- | --- |
| **HL10** | NS | I-III | NEG | F | YOUNG | WOMAN | LOW | ABVDx6 | CR | NO |
| **HL12** | OTHER | I-III | NEG | U | YOUNG | WOMAN | LOW | ABVDx6 + Rt | PD | NO |
| **HL13** | OTHER | I-III | POS | U | YOUNG | MAN | LOW | ABVDx6 | PD | NO |
| **HL14** | OTHER | IV | NEG | F | OLD | MAN | HIGH | ABVDx6 | CR | YES |
| **HL15** | NS | I-III | NEG | U | YOUNG | MAN | LOW | ABVDx6 + Rt | CR | YES |
| **HL17** | NS | I-III | NEG | U | YOUNG | MAN | NA | ABVDx4 | PD | NO |
| **HL19** | NA | I-III | NA | F | YOUNG | NA | NA | OEPAx2 + COPDACx2 + Rt | CR | YES |
| **HL2** | OTHER | IV | POS | F | OLD | WOMAN | HIGH | BEACOPP | CR | YES |
| **HL20** | NA | IV | NEG | U | YOUNG | MAN | HIGH | ABVDx6 | PD | NO |
| **HL21** | OTHER | IV | POS | U | OLD | MAN | NA | ABVDx6 | PD | NO |
| **HL22** | OTHER | I-III | NEG | U | YOUNG | MAN | HIGH | ABVDx6 | PD | NO |
| **HL23** | NS | IV | NEG | U | YOUNG | WOMAN | LOW | ABVDx6 | PD | NO |
| **HL24** | NS | I-III | NEG | U | YOUNG | MAN | LOW | ABVDx6 | CR | YES |
| **HL25** | NS | I-III | NEG | F | YOUNG | WOMAN | NA | ABVDx6 | CR | NO |
| **HL28** | NS | IV | NEG | U | YOUNG | MAN | LOW | ABVDx6 | PD | NO |
| **HL29** | NS | IV | NA | U | YOUNG | MAN | LOW | No data | PR | NO |
| **HL3** | OTHER | IV | NEG | F | YOUNG | MAN | HIGH | BEACOPP | CR | NO |
| **HL4** | OTHER | I-III | NEG | F | YOUNG | WOMAN | LOW | ABVDx6 | CR | NO |
| **HL40** | OTHER | I-III | NEG | F | YOUNG | WOMAN | LOW | ABVDx6 + Rt | CR | NO |
| **HL41** | OTHER | I-III | POS | F | YOUNG | WOMAN | LOW | ABVDx6 | CR | NO |
| **HL47** | OTHER | I-III | POS | F | OLD | MAN | NA | ABVDx6 | CR | YES |
| **HL5** | OTHER | IV | NEG | U | YOUNG | MAN | HIGH | ABVDx6 | PD | NO |
| **HL6** | NS | I-III | NEG | F | YOUNG | WOMAN | LOW | ABVDx6 | CR | NO |
| **HL8** | NS | I-III | NEG | U | YOUNG | MAN | LOW | ABVDx6 | PR | NO |
| **HL9** | NS | IV | POS | U | YOUNG | WOMAN | LOW | ABVDx6 | PR | NO |

NS = nodular sclerosis; LR = lymphocyte-rich; MC = mixed cellularity; LD = lymphocytic depletion; IPS = International Prognostic Score; ABVD = adriamycin, bleomycin, vinblastine, and dacarbazine; Rt = rituximab; CR = complete remission; PD = progressive disease; PR = partial response; OTHER = other subtypes (LR, MC, or LD); NA = no data; BEACOPP = bleomycin, etoposide, adriamycin, cyclophosphamide, vincristine, procarbazine, and prednisone; OEPA = vincristine, etoposide, prednisone, and adriamycin; COPDAC = cyclophosphamide, vincristine, prednisone, and dacarbazine; OLD = at least 65 years old; YOUNG = younger than 65 years; IV = stage IV; OS = other stages; POS = infected with Epstein–Barr virus; NEG = not infected; YES = relapse; NO = no relapse; U = unfavorable; F = favorable (cases considered to be F if complete response after first line of treatment, maintained for at least two years; cases designated U otherwise).


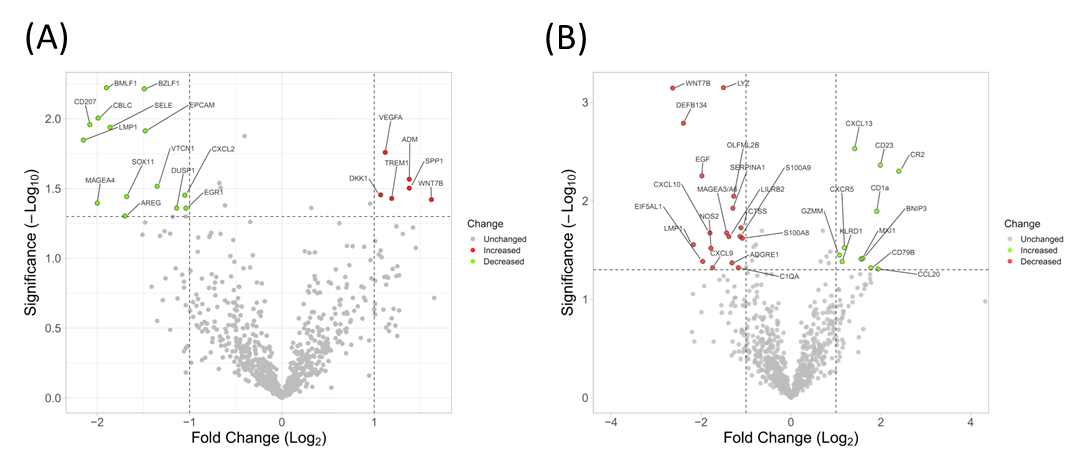


**Supplemental Figure 1.** **Differential expression of genes between groups.** (A) Volcano plot showing genes upregulated or downregulated between favorable (left, green) and unfavorable (right, red) cHL patients (absolute log_2_-fold change >1 and a significant FDR (<0.05)). (B) Volcano plot showing differential expressed genes between relapsed (left, red) and non-relapsed (right, green) cHL patients (absolute log_2_-fold change >1 and a significant FDR (<0.05)). Both were generated using the VolcaNoseR open-source website (<https://huygens.science.uva.nl/VolcaNoseR/>).


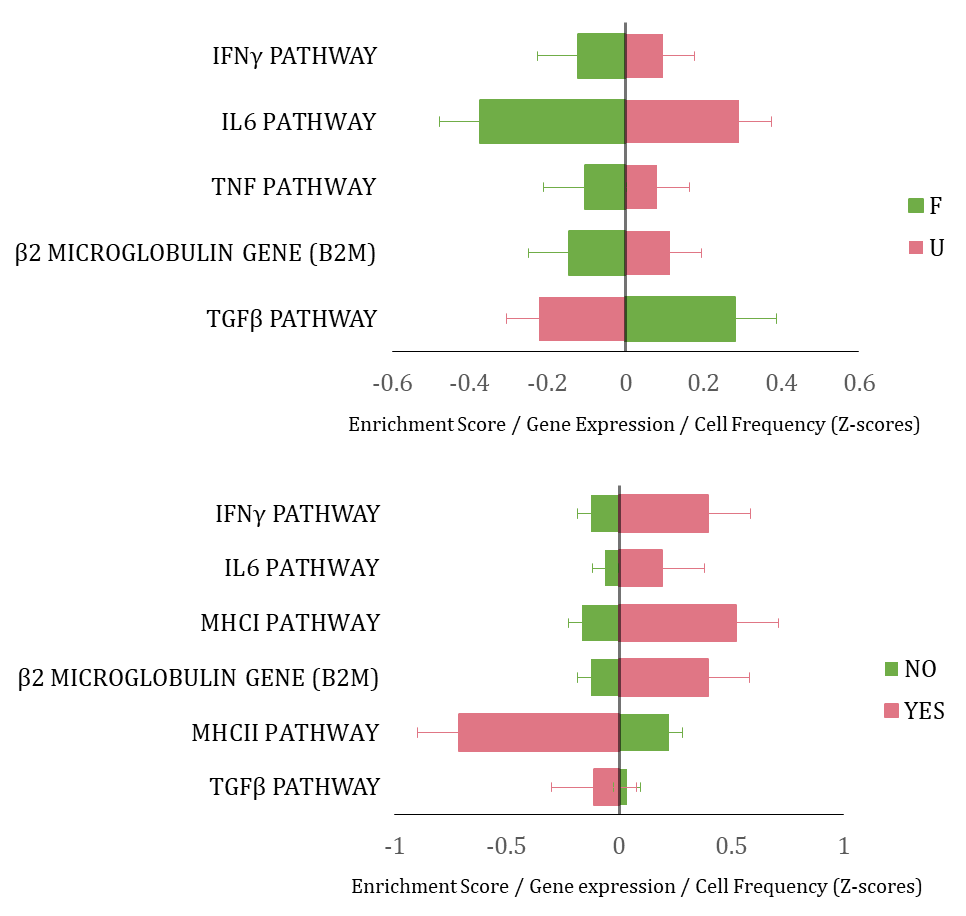


**Supplemental Figure 2.** **Bar graphs of inflammation balance and MHC molecules.** Above, comparison of enrichment scores, gene-expression values, or cell frequencies in favorable and unfavorable patient groups. Below, comparison of patients who relapsed (YES) and who did not (NO). Values are represented as Z-scores for ease of comparison (mean subtracted from value, divided by standard deviation of that element). Variables are enriched in the group of patients with a positive score.


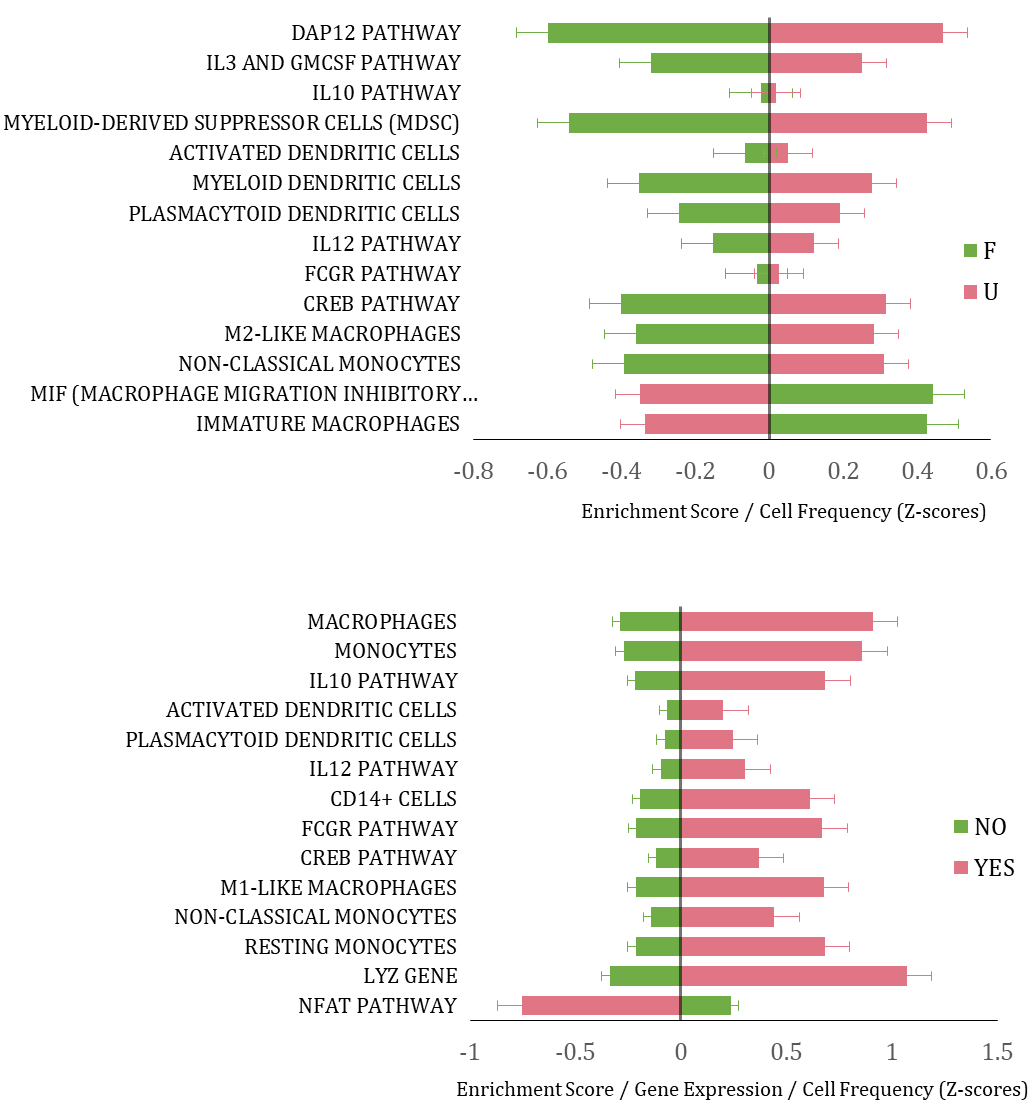


**Supplemental Figure 3.** **Bar graphs of myeloid cells.** Above, comparison of enrichment scores, gene-expression values, and cell frequencies for favorable and unfavorable patient groups. Below, comparison of patients who relapsed (YES) and who did not (NO). Values are represented as Z-scores for ease of comparison (mean subtracted from value, divided by standard deviation of that element). Variables are enriched in the group of patients with a positive score.


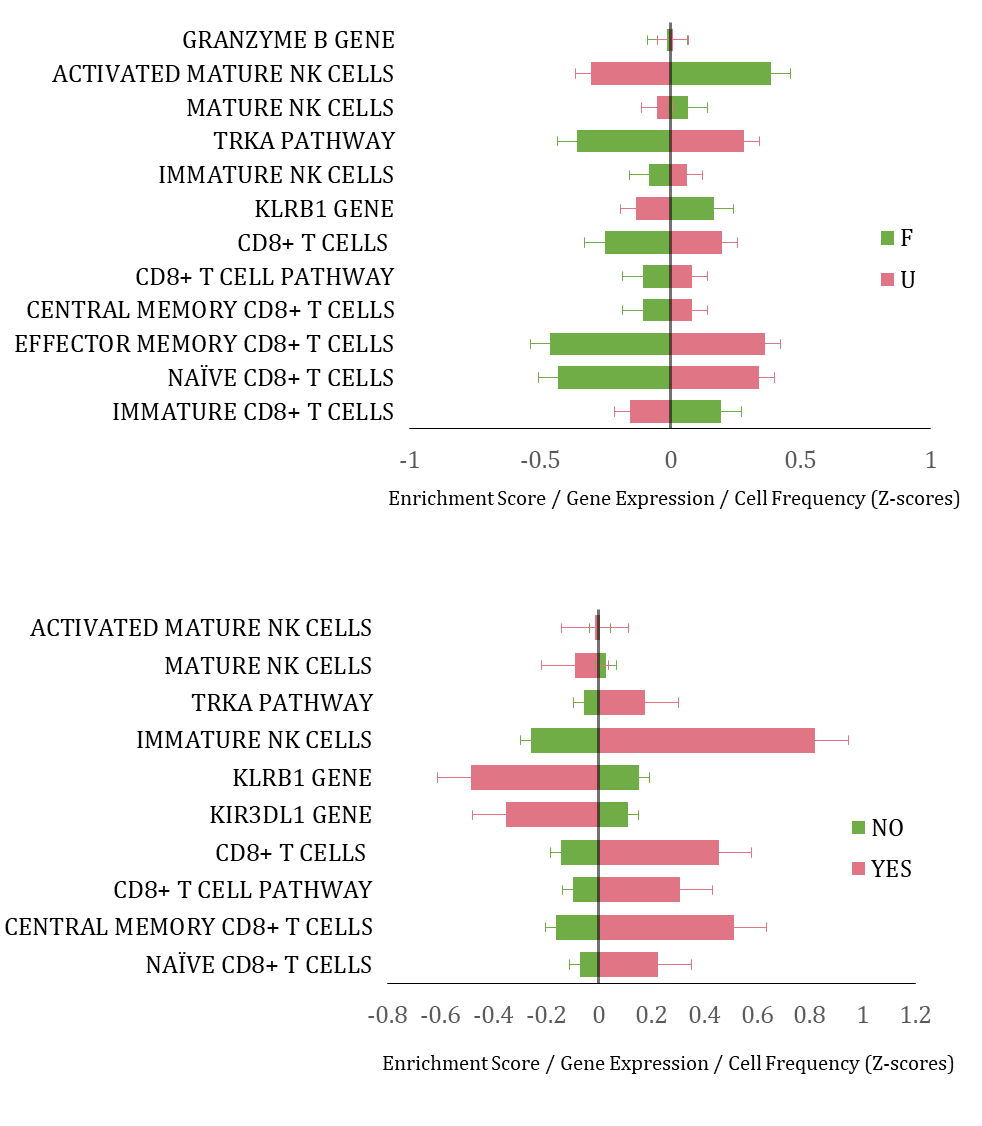


**Supplemental Figure 4.** **Bar graphs for cytotoxic cells.** Above, comparison of enrichment scores, gene-expression values, and cell frequency in favorable and unfavorable patients. Below, comparison of patients who relapse (YES) and who do not (NO). Values are represented as Z-scores for ease of comparison (mean subtracted from value, divided by standard deviation of that element). Variables are enriched in the group of patients with a positive score.


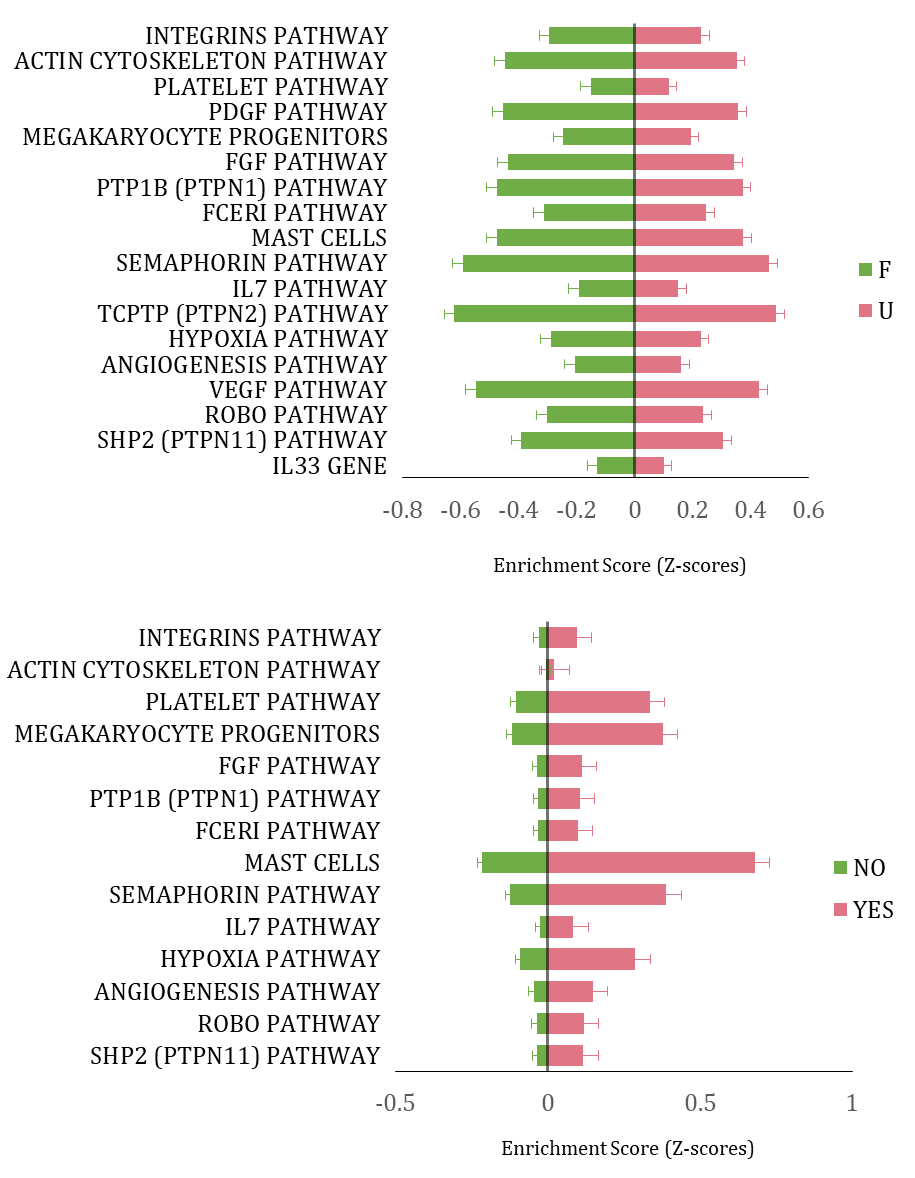


**Supplemental Figure 5.** **Bar graphs of stromal components.** Above, comparison of enrichment scores, gene-expression values, and cell frequencies between favorable and unfavorable patients. Below, comparison of patients who relapsed (YES) and who did not (NO). Values are represented as Z-scores for ease of comparison (mean subtracted from value, divided by the standard deviation of that element). Variables are enriched in the group of patients with a positive score.


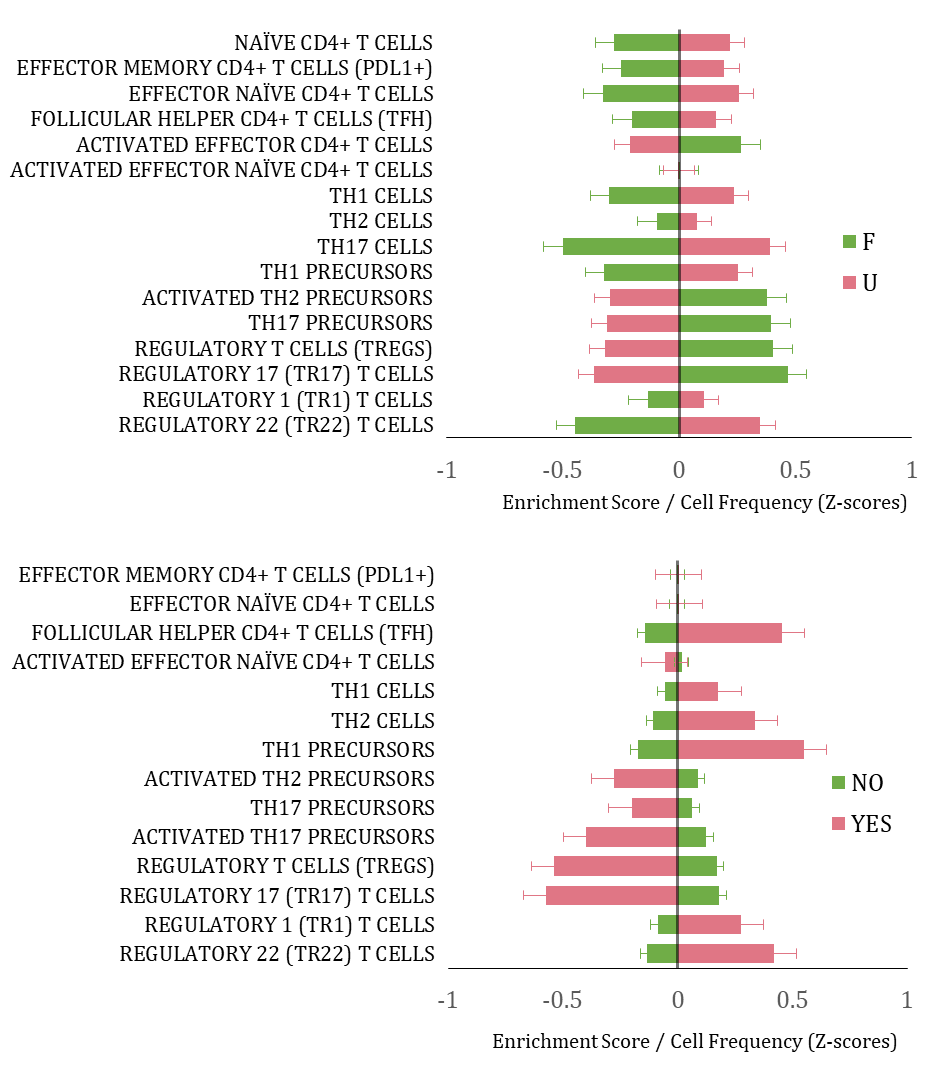


**Supplemental Figure 6.** **Bar graphs of CD4+ T cells.** Above, comparison of enrichment scores, gene-expression values, and cell frequencies of favorable and unfavorable patients. Below, comparison of patients who relapsed (YES) and who did not (NO). Values are represented as Z-scores for ease of comparison (mean subtracted from value, divided by standard deviation of that element). Variables are enriched in the group of patients with a positive score.


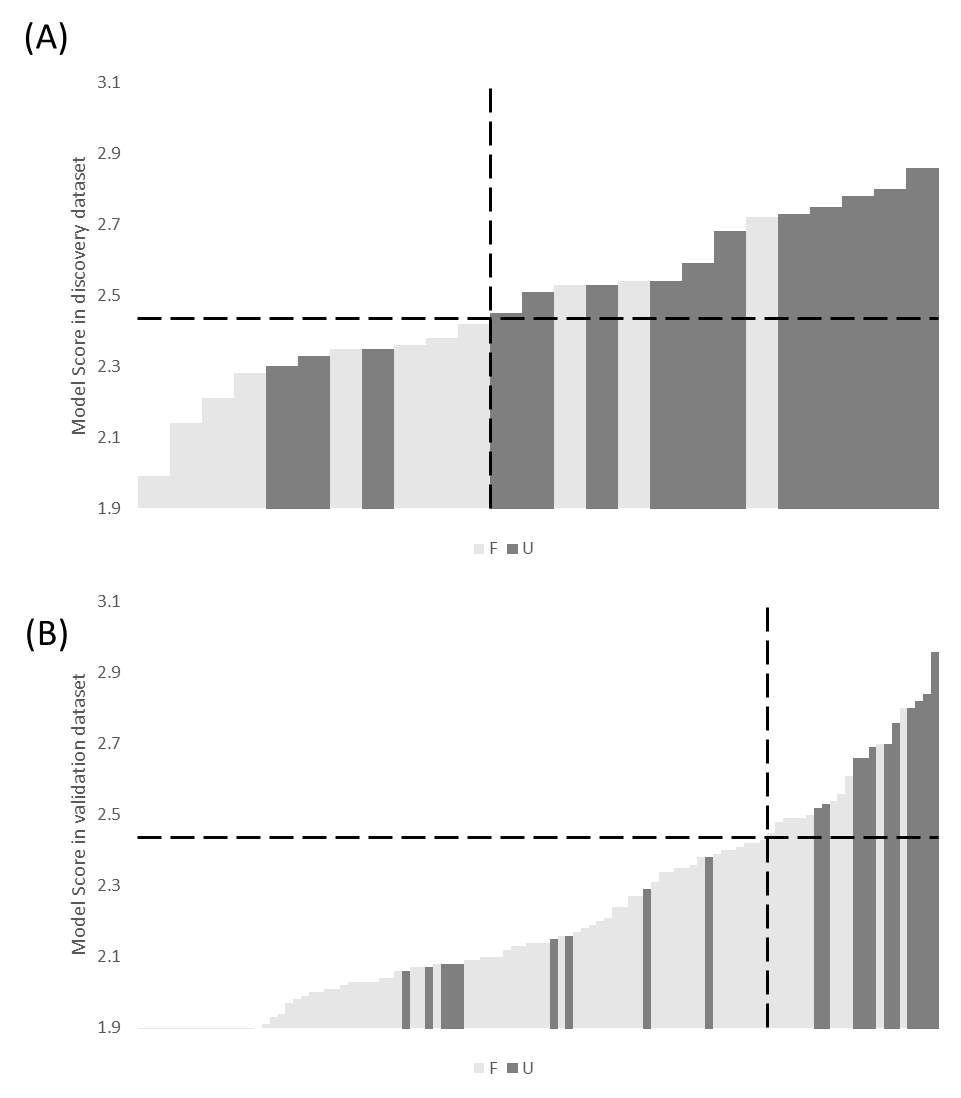


**Supplemental Figure 7.** **Representation of model scores for discovery and validation datasets.** Each column represents a single patient from the discovery (A) or validation (B) cohort, arranged in ascending order of score (left to right). The vertical dotted line divides patients into low- and high-risk groups according to the threshold predictor score, which was determined in the discovery cohort using ROC curves.
